# Polymer Dots as Photoactive Membrane Vesicles for [FeFe]-Hydrogenase Self-Assembly and Solar-Driven Hydrogen Evolution

**DOI:** 10.1021/jacs.2c03882

**Published:** 2022-07-21

**Authors:** Mariia V. Pavliuk, Marco Lorenzi, Dustin R. Morado, Lars Gedda, Sina Wrede, Sara H. Mejias, Aijie Liu, Moritz Senger, Starla Glover, Katarina Edwards, Gustav Berggren, Haining Tian

**Affiliations:** † Department of Chemistry—Ångström Laboratory, Physical Chemistry, Uppsala University, 751 20 Uppsala, Sweden; ‡ Department of Chemistry—Ångström Laboratory, Molecular Biomimetics, Uppsala University, 751 20 Uppsala, Sweden; § Department of Biochemistry and Biophysics, Science for Life Laboratory, Stockholm University, 171 65 Solna, Sweden

## Abstract

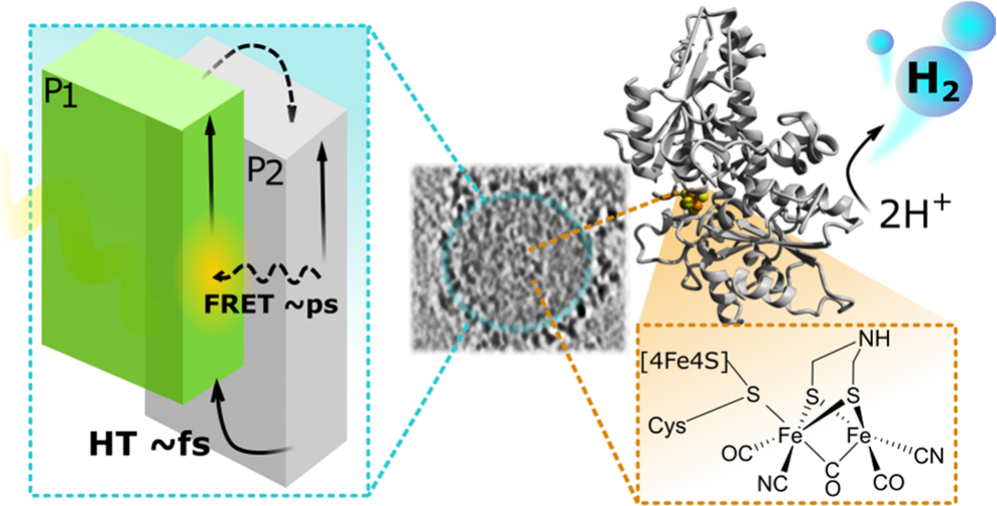

A semiartificial photosynthesis approach that utilizes enzymes for solar fuel production relies on efficient photosensitizers that should match the enzyme activity and enable long-term stability. Polymer dots (Pdots) are biocompatible photosensitizers that are stable at pH 7 and have a readily modifiable surface morphology. Therefore, Pdots can be considered potential photosensitizers to drive such enzyme-based systems for solar fuel formation. This work introduces and unveils in detail the interaction within the biohybrid assembly composed of binary Pdots and the HydA1 [FeFe]-hydrogenase from *Chlamydomonas reinhardtii*. The direct attachment of hydrogenase on the surface of toroid-shaped Pdots was confirmed by agarose gel electrophoresis, cryogenic transmission electron microscopy (Cryo-TEM), and cryogenic electron tomography (Cryo-ET). Ultrafast transient spectroscopic techniques were used to characterize photoinduced excitation and dissociation into charges within Pdots. The study reveals that implementation of a donor–acceptor architecture for heterojunction Pdots leads to efficient subpicosecond charge separation and thus enhances hydrogen evolution (88 460 μmol_H2_·g_H2ase_^–1^·h^–1^). Adsorption of [FeFe]-hydrogenase onto Pdots resulted in a stable biohybrid assembly, where hydrogen production persisted for days, reaching a TON of 37 500 ± 1290 in the presence of a redox mediator. This work represents an example of a homogeneous biohybrid system combining polymer nanoparticles and an enzyme. Detailed spectroscopic studies provide a mechanistic understanding of light harvesting, charge separation, and transport studied, which is essential for building semiartificial photosynthetic systems with efficiencies beyond natural and artificial systems.

## Introduction

Semiartificial photosynthesis is a promising strategy for renewable fuel formation as it merges the efficiencies of biocatalysis with the outstanding properties of synthetic photosensitizers.^1^ This approach has spurred the engineering of biohybrid systems, where enhanced solar fuel production can be achieved by the synergetic effect of the catalytic power of highly active and selective enzymes for proton reduction, namely, hydrogenases (H_2_ases), and efficient light harvesters.

To date, several types of biohybrid systems have been examined including assemblies where enzymes are immobilized on electrodes (carbon nanotubes,^2−5^ redox hydrogels,^6,7^ Au,^8,9^ TiO_2_)^10^ or form complexes with photoactive materials (carbon dots,^11,12^ CdS nanorods,^13^ inorganic semiconductors).^14−16^ In these systems, the interface between the enzyme and the material plays an important role in the interfacial charge transfer and recombination reactions. A clear design approach should include the fine tuning of interactions between the light-harvesting and catalytic units to match energy levels and optimize the energy flow in the desired direction.^14^ This highlights the importance of finding a proper photosensitizer to match the enzyme’s performance. Light harvesters, which have dominated the field of semiartificial photosynthesis, are known to suffer from high toxicity (e.g., Cd-based materials),^14,15,17^ low dispersibility in aqueous media (e.g., TiO_2_),^16,18^ and poor visible light absorption of the solar spectrum with some exceptions.^19^ Metal-free graphitic carbon nitrides offer stability but have a narrow light-harvesting region and exhibit weak enzyme interactions.^20^ The development of biohybrid complexes needs light-harvesting materials that meet the following requirements: (1) have flexible morphology with ligands or surface groups that will promote efficient binding and maintenance of the enzyme within close proximity to minimize the electron diffusion distance (preferably via noncovalent forces, e.g., electrostatic or hydrophobic interaction), (2) are compatible with the size of the enzyme (e.g., have pores greater than the enzyme for efficient encapsulation), (3) provide efficient charge separation, accumulation, and transport, and (4) minimize charge recombination.

Among photoactive materials that fulfill the requirements for biohybrid assemblies, polymer dots (Pdots) have emerged as efficient biocompatible^21^ nanoparticles with highly tunable optical properties,^22−28^ diverse chemical structures,^29−32^ stability,^33^ and amenability to surface morphological and functional group alterations.^34^ Pdots of smaller sizes (<100 nm) not only have a large surface area but also facilitate efficient charge separation at the interface to eliminate the limits set by short exciton diffusion lengths (5–20 nm),^35,36^ thus enhancing the movement of excitons toward the surface of the Pdots and further to the catalytic active site. Implementation of charged polymer side chains can further facilitate the interaction with an oppositely charged catalyst precursor, resulting in close interaction and thus enhanced hydrogen evolution. In contrast to inorganic semiconductors, where typically high dielectric constants and low exciton binding energies (∼10 meV) favor charge separation to free charge carriers upon photoexcitation, organic semiconductors are characterized by low dielectric constants and high exciton binding energies, thus generating upon photoexcitation electrostatically bound excited electron and hole pairs (excitons) that are less prone to be separated. Inspired by the organic solar cell field, this long-standing problem of initially poor charge separation in organic materials can be suppressed by preparing Pdots (or polymer nanoparticles as well) with electron donor (D)/electron acceptor (A) heterojunctions.^37−39^ Precise selection of energy levels for D/A polymers results in directing the electron through an energetically downhill path with minimal losses via the successive steps of energy and charge transfer.^22^ To the best of our knowledge, in spite of advantageous physicochemical properties offered by Pdots, no biohybrid assemblies with enzymes that are photocatalytically active for fuel production have been presented to date.

In this study, we introduce the biohybrid assembly of heterojunction Pdots with the HydA1 [FeFe]-hydrogenase from *Chlamydomonas reinhardtii* for photocatalytic hydrogen production.^40−43^ The interactions and electron transfer between the subunits that lead to efficient solar-driven hydrogen evolution are examined in detail.

## Experimental Section

### Materials

Polymer poly(9,9-dioctylfluorene-*alt*-bithiophene), also known as F8T2 (coded as **P1** in this work, *M*_w_ 64 kDa), was purchased from Ossila, U.K. Triblock copolymer (**P2**, *M*_w_ 20–30 kDa) poly(*N*,*N*-dimethylamino ethyl methacrylate)-B-poly(9,9-*N*-dihyxyl-2,7-fluorene)-B-poly(*N*,*N*-dimethylamino ethyl methacrylate) and copolymer polystyrene grafted with carboxy-terminated polyethylene oxide (PS-PEG-COOH, backbone chain *M*_w_ 8.5 kDa, graft chain *M*_w_ 4.6 kDa, total chain 36.5 kDa) were purchased from Polymer Source Inc., Canada. The hydrogenase CrHydA1 (*M*_w_ 50 kDa) was prepared following literature protocols, and the specific activity was 325 μmol_H2_·mg_H2ase_^–1^·min^–1^.^44,45^ In Supporting Information (SI) details about hydrogenase expression, purification, reconstitution, and maturation are presented. Centrifugal filters with molecular weight (*M*_w_) cutoffs of 10 kDa (Amicon Ultra-15) and 100 kDa (Vivaspin 2) were purchased from Merck and Sigma-Aldrich, respectively. Tetrahydrofuran (THF) and absolute ethanol (99.5%+) were purchased from VWR Chemicals. Other chemicals were obtained from Sigma-Aldrich and all chemicals were used without further purification unless stated otherwise.

### Preparation of Pdots

Pdots were prepared by a modified nanoprecipitation method.^32,46^ P1 and PS-PEG-COOH were dissolved in THF at a concentration of 1 mg·mL^–1^ and then sonicated for 30 min. P1 solution (2 mL, 1 mg·mL^–1^) and triblock copolymer P2 (10 mg) were mixed in 10 mL of THF to create a final weight ratio between components of 1:5, and then 100 μL of PS-PEG-COOH (1 mg·mL^–1^) was added. Furthermore, this mixture was sonicated for additional 30 min under argon stripping to ensure fine distribution of all components in the organic phase. The resulting dark yellow solution was slowly added dropwise into 20 mL of distilled water and left under argon purging for additional 30 min. THF was removed by slow evaporation. The resulting aqueous polymer solution was passed through a 0.45 μm syringe filter and further purified by size exclusion chromatography; 500 μL of Pdots were eluted with water on a Sepharose CL-4B column at a flow rate of 0.5 mL·min^–1^. The polymer concentration in the final mixture was estimated by the analysis of UV–vis spectra of freeze-dried Pdots redissolved in THF. Pdots composed of one light-harvesting polymer (either P1 or P2) were prepared following the procedure specified above.

### Gel Electrophoresis

Agarose gels were prepared according to the standard protocol by mixing 0.8 g of agarose with 100 mL of 1× TAE buffer (40 mM Tris, 20 mM acetic acid, and 1 mM EDTA) and heating the solution until agarose was completely dissolved. The melted 0.8% agarose solution was poured into a gel mold with a well comb in place to cure at room temperature for 30 min. The solidified gel was placed into the electrophoresis cell along with 1× TAE buffer.

Sample preparation for gel electrophoresis was as follows: Pdots (125 ng·mL^–1^), hydrogenase (75 μM), TEOA (5 μL 10 vol %, pH 7), and loading buffer (5 μL, stock: 8 mg bromophenol blue, 4 mL glycerol, 5 mL 0.5 M Tris, pH 7) were mixed with a Hamilton syringe and left for incubation for 1 h inside a glovebox with a humid Ar atmosphere (total sample volume of 20 μL).

The three samples of Pdots, hydrogenase, and Pdots loaded with hydrogenase were added to three wells of the gel. An applied voltage of 150 V was maintained during electrophoresis. The gel was imaged using a Spectroline Ultraviolet transilluminator (Techtum lab). Hydrogenase was visualized by the Coomassie Blue staining method.

### Cryo-Transmission Electron Microscopy (Cryo-TEM)

Cryo-TEM images were captured using a Zeiss Libra 120 transmission electron microscope (Carl Zeiss AG, Oberkochen, Germany) operating at 80 kV and in zero-loss bright-field mode. Digital images were recorded under low-dose conditions with a BioVision Pro-SM Slow Scan CCD camera (Proscan Elektronische Systeme GmbH, Scheuring, Germany). Pdots samples (0.6 mg·mL^–1^) were incubated with hydrogenase (3.2 μM) for 12 h. All unbound hydrogenase was removed by centrifugal filtration with an *M*_w_ cutoff of 100 kDa. Samples were equilibrated at 25 °C and high relative humidity within a climate chamber and analyzed as described earlier.^47^ A small drop of each sample was deposited on a carbon-sputtered copper grid covered with a perforated polymer film. Excess liquid was thereafter removed by blotting with a filter paper, leaving a thin film of the solution on the grid. The sample was vitrified in liquid ethane and transferred to the microscope, continuously kept below −160 °C, and protected against atmospheric conditions.

### Cryo-Transmission Electron Tomography (Cryo-ET)

R3.5/1 200 mesh grids (QuantiFoil) were glow-discharged (20 mA 60 s) on a PELCO EasiGlow. Pdots samples (0.6 mg·mL^–1^) were incubated with hydrogenase (4.1 μM) for 1 h inside the glovebox; 10 nm protein-A gold fiducials (Aurion) were resuspended and mixed properly with samples containing bare Pdots or Pdots with hydrogenase at 2:3 and 1:4 ratios, respectively. Three microliters of this mixture was applied onto grids before plunge-freezing into liquid ethane in a Vitroblot Mark IV robot (FEI/Thermo Fisher Scientific) operating at 4 °C, 100% humidity, and with a blot time of 3 s. Data sets were collected using a Titan Krios G3i microscope (FEI/Thermo Fisher Scientific) outfitted with a K3 detector and a BioQuantum imaging filter (Gatan) operating at 300 kV in the nanoprobe and EF-TEM mode with a C2 aperture size of 50 μm, an objective aperture size of 70 μm, and an energy filter slit width of 20 eV. Tomo5 software (Thermo Fisher Scientific) was used to acquire each tilt series using a bidirectional tilt scheme with a range of ±60°, 2° angular increase, and a target defocus of −3 to −6 μm. Each tilt series of 61 7-frame movies was recorded in the counting mode with a pixel size of 1.67 Å at a dose rate of 15 e^–^/px/s for 0.373 s and a total dose per tilt series of ∼122 e^–^/Å^2^. The initial raw movies were aligned and dose-weight filtered using “alignframes” from the IMOD package.^48^ Tilt series were aligned using gold fiducial markers, the contrast transfer function (CTF) was estimated, and bin2 (3.34 Å/px) tomograms were reconstructed by weighted back-projection with three-dimensional (3D)-CTF correction by phase flipping using programs within the IMOD package.^49,50^ Tomograms were denoised using Topaz-Denoise and further downsampled to a 6.68 Å pixel size for analysis.^51^

### Dynamic Light Scattering (DLS) and Surface Zeta Potential Measurements

The hydrodynamic diameter and surface ξ-potentials of the samples were measured on a Zetasizer Nano-ZS (Malvern, U.K.) in quartz and folded capillary zeta cells, respectively.

### NMR Characterization

The NMR spectra of polymers used for Pdots preparation were recorded in THF-*d*_8_ on a JEOL resonance 400 MHz spectrometer.

### Steady-State Absorption and Photoluminescence (PL) Measurements

UV–vis spectra were measured on a Varian Cary 5000. Steady-state PL spectra were acquired on a Fluorolog iHR 320 (Horiba Jobin Yvon) using the right-angle mode and monochromatic narrow-band excitation in a quartz cuvette. The photoemission quantum yields (QYs) were estimated according to the standard procedure using Coumarin 343 as a reference dye (QY = 63%).^52,53^

### Transient Absorption Spectroscopy (TAS)

The output from a Coherent Libra Ti:Sapphire amplifier (1.5 mJ, 3 kHz, FWHM 45 fs) was split into a pump and a probe. The 398 nm excitation light was obtained by doubling the frequency of the fundamental light (796 nm), while the 490 nm excitation light was obtained by directing the pump beam into the optical parametric amplifier, TOPAS-White/TOPAS-Prime, and TOPAS SHS/TOPAS NirVis (Light Conversion). The output was passed through a mechanical chopper, blocking every second pulse, and was focused onto the sample. The pump intensity was attenuated to 200 μW. The white-light probe (330–750 nm) was obtained by focusing part of the fundamental 796 nm light on a moving CaF_2_ crystal (Newport TAS). Both pump and probe lights were redirected to the Newport MS260i spectrograph with interchangeable gratings. The probe spectrum was recorded on a silicon diode array (custom made, Newport). The instrumental response time was typically around 150–180 fs. The transient absorption spectra at different times were recorded by delaying the probe beam relative to the pump from −15 ps to 8 ns with the help of an optical delay line. For each sample, four scans were collected and averaged. SurfaceXplorer was used to autocorrect for pump scattering. The kinetic traces were fitted as a sum of convoluted exponentials. TA kinetic traces on a ns-to-s timescale were measured using a ns-laser pump–probe setup,^54^ where the samples were excited with a Nd:YAG laser (Quantel, BrilliantB) that delivered 25 mJ/pulse at 355 nm. To minimize sample excitation by probe light, this light was passed through a double monochromator setup (Applied Photophysics, pbp Spectra Kinetic Monochromator 05–109) with 4- and 2-mm slit openings before and after the sample, respectively. The signal was detected by a photomultiplier tube (PMT, Hamamatsu R928) and further digitized using an Agilent Technologies Infiniium digital oscilloscope (600 MHz). TA traces were processed with Applied Photophysics LKS software. Pdots samples without and with hydrogenase were prepared in quartz cuvettes (1 mm × 10 mm path length for fs TA, and 4 mm × 10 mm path length for ns TA) inside an oxygen-free glovebox.

### Time-Resolved Fluorescence Measurements

Excitation of the Pdots samples was performed with pump pulses generated via an optical parametric amplifier from 800 nm pulses, which were converted to 740 nm and subsequently doubled to attain 370 nm with an OPO crystal. Aqueous solutions of Pdots were measured using a 1 cm quartz cuvette and an excitation power at the sample of 300 μW. Fluorescence at a right angle to the excitation was passed through a Bruker SPEC 250IS spectrograph (ca. 200 nm observation window) and onto the streak camera (Hamamatsu streak camera and blanking unit C5680 in combination with a Synchroscan Unit M5675). A charge-coupled device (CCD) camera (Hamamatsu Orca-ER C4742–95) was used in the binning mode (2 × 2 pixels) to give a 512 × 512 pixel matrix. The observed time window in time range 2 was 700 ps, while in time range 4, it was 2000 ps with FWHM instrument response functions of 30 or 45 ps. For the decay curves obtained from the streak camera, the procedure written in Matlab was employed assuming a Gaussian instrument response function. Time-correlated single photon counting (TCSPC) measurements were carried out in a time window of 50 ns using a pulsed diode laser source (Edinburgh Instruments EPL470, λ_exc._ = 405 nm).^32^

### Spectroelectrochemistry

Spectroelectrochemistry was performed in a quartz cuvette with a 1 cm path length. Cyclic voltammograms were carried out using an Autolab potentiostat (PGSTA302) with a GPES electrochemistry interface (Eco Chemie). A three-electrode cell was composed of a Pt wire as a counter electrode, Ag/AgNO_3_ (0.1 M AgNO_3_ in acetonitrile) as a reference electrode, and polymer-coated fluorine-doped tin oxide (FTO) glass as a working electrode in acetonitrile with tetrabutylammonium hexafluorophosphate (TBAPF_6_, previously dried at 80 °C in a vacuum oven) as the supporting electrolyte (0.1 M). Ferrocene/ferrocenium (*F*c/*F*c^+^) was used as an internal reference with a potential versus the normal hydrogen electrode (NHE) value of +0.63 V vs NHE.^55^ Both P1 and P2 (6 mg·mL^–1^ in CHCl_3_) were spin-coated on FTO substrates to form uniform films (1000 rpm, 30 s). Before all measurements, oxygen was removed from the cell by bubbling solvent-saturated argon through the solution. UV–vis/NIR spectra of the reduced and oxidized polymer species were recorded with a Hewlett Packard diode array spectrometer (model 8453) during electrochemical experiments. When possible, the concentration was adjusted to give an absorbance of 0.6 at the excitation wavelength. The excited-state potentials (*E*_P1^–^/P1*_, *E*_P2^–^/P2*_) were determined by subtraction of 0-0 transition energy (*E*_0–0_) from the peak reduction potential of the polymer (*E*_P1^–^/P1_, *E*_P2^–^/P2_; eqs 1 and 2). *E*_0–0_ was determined from the intersection of the normalized absorption and photoluminescence spectra.
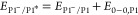
1
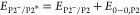
2

### Evaluation of Photocatalytic Activity

The photocatalytic hydrogen evolution was measured in 9 mL gastight vials. Pdots (1–50 μg·mL^–1^) and hydrogenase (17.5–600 pmol·mL^–1^), were mixed inside a glovebox with a humid Ar atmosphere. Methyl viologen (5–25 mM) and triethanolamine (TEOA, from 10 vol %, 0.67 M to 30 vol %, 2 M; pH 7.0) or ethylenediaminetetraacetic acid (EDTA, 0.1 M, pH 7.0) were added to the resulting mixture to give a 2 mL sample volume. Pdots samples were illuminated with an LED PAR38 lamp (17 W, 50 mW·cm^–2^, 420–750 nm) that had a similar light intensity between 420 and 750 nm of 1 Sun—100 mW·cm^–2^. The amount of hydrogen evolved was determined by gas chromatography (PerkinElmer LLC, MA) from the calibration curve obtained for the known quantities of injected hydrogen. After each removal of hydrogen from the headspace, the Hamilton needle and the top of the vial septa were covered with Play-Doh clay (Hasbro, Inc) before the needle was taken out to minimize oxygen contamination. The experiments were performed in triplicate to obtain standard deviations. The corresponding data were presented in the form of both evolved hydrogen per gram of hydrogenase and per moles of hydrogenase (TON).

The external quantum efficiency (EQE) was calculated according to eq 3.
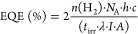
3where *n*(H_2_) is the moles of photogenerated hydrogen, *N*_A_ is the Avogadro constant, *h* is the Planck constant, *c* is the speed of light, λ is the excitation wavelength, *t*_irr_ is the irradiation time, *I* is the intensity of illumination, and *A* is the irradiated area. Measurements were performed in a vial containing 2 mL of solution, namely, Pdots (10 μg·mL^–1^), CrHydA1 (75 pmol·mL^–1^), methyl viologen (MV^2+^, 5 mM), and TEOA (30 vol %, pH 7). Samples were illuminated with a 405 nm monochromatic laser (*d* = 1.5 cm) or a Xe lamp (300 W, AULTT CEL-HXF300 / CEL-HXUV300) equipped with an AM1.5 filter and a bandpass filter (CEAULIGHT, 420 nm).

## Results and Discussion

### Design and Preparation of the Pdots

**Figure 1:**
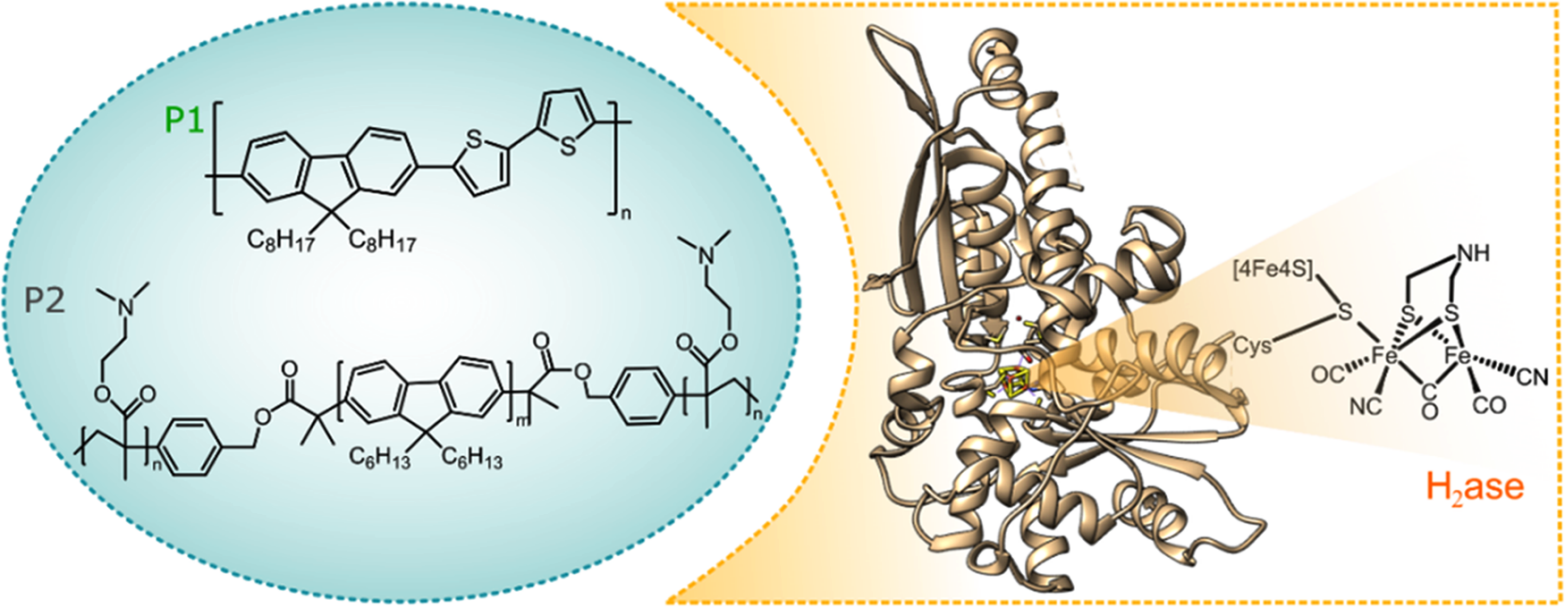
Structures of polymers used in the preparation of Pdots, and a model of HydA1 [FeFe]-hydrogenase generated from its crystal structure (PDB ID: 3LX4). The active site of hydrogenase (H-cluster) is schematically represented.

The Pdots surface provides a versatile platform where the polymer can be designed to optimize the interaction at the polymer enzyme interface. For this study, Pdots are prepared via the nanoprecipitation method,^56^ employing two polymers: F8T2 as the donor polymer (P1) and poly(*N*,*N*-dimethylamino ethyl methacrylate)-B-poly(9,9-*N*-dihyxyl-2,7-fluorene)-B-poly-(*N*,*N*-dimethylamino ethyl methacrylate) as the acceptor polymer (P2, Figure 1). Pdots were purified (Figure S1 in the SI) and had an average hydrodynamic diameter of 50–70 nm as determined by dynamic light scattering (DLS, Figure S2). The mass ratio between polymers P1 and P2 in the final product was 1:3 as estimated from the corresponding calibration curves (Figure S3). Powder X-ray Diffraction (PXRD) measurements revealed that binary P1/P2 Pdots were (semi)crystalline predominantly due to P2 polymer (Figure S4). Implementation of polymers with phase separation is known to facilitate fast charge generation by omitting losses due to diffusion of the excitons to the interface,^56^ thus suppressing unwanted geminate recombination.^57,58^ Both P1 and P2 have long alkyl chains to facilitate hydrophobic interactions with the enzyme (Figure 1). At the same time on both sides of the polyfluorene unit of P2 additional branched copolymers with tertiary amine-terminated groups were grafted for further facilitation of electrostatic interaction with the enzyme (Figures S5 and S6). Zeta potential (ξ) measurements were performed to investigate the surface charge of Pdots (Table S1). Under photocatalytic conditions (pH 7), binary P1/P2 Pdots are positively charged (ξ = +31 mV), which is most likely due to the protonation of tertiary amine-terminated surface groups (Pdots–NHMe_2_^+^) and can therefore interact with the hydrogenase surface, which has a negative net surface charge (isoelectric point, pI = 5.86).

### Visualizing the Pdots:H_2_ase Interaction

**Figure 2:**
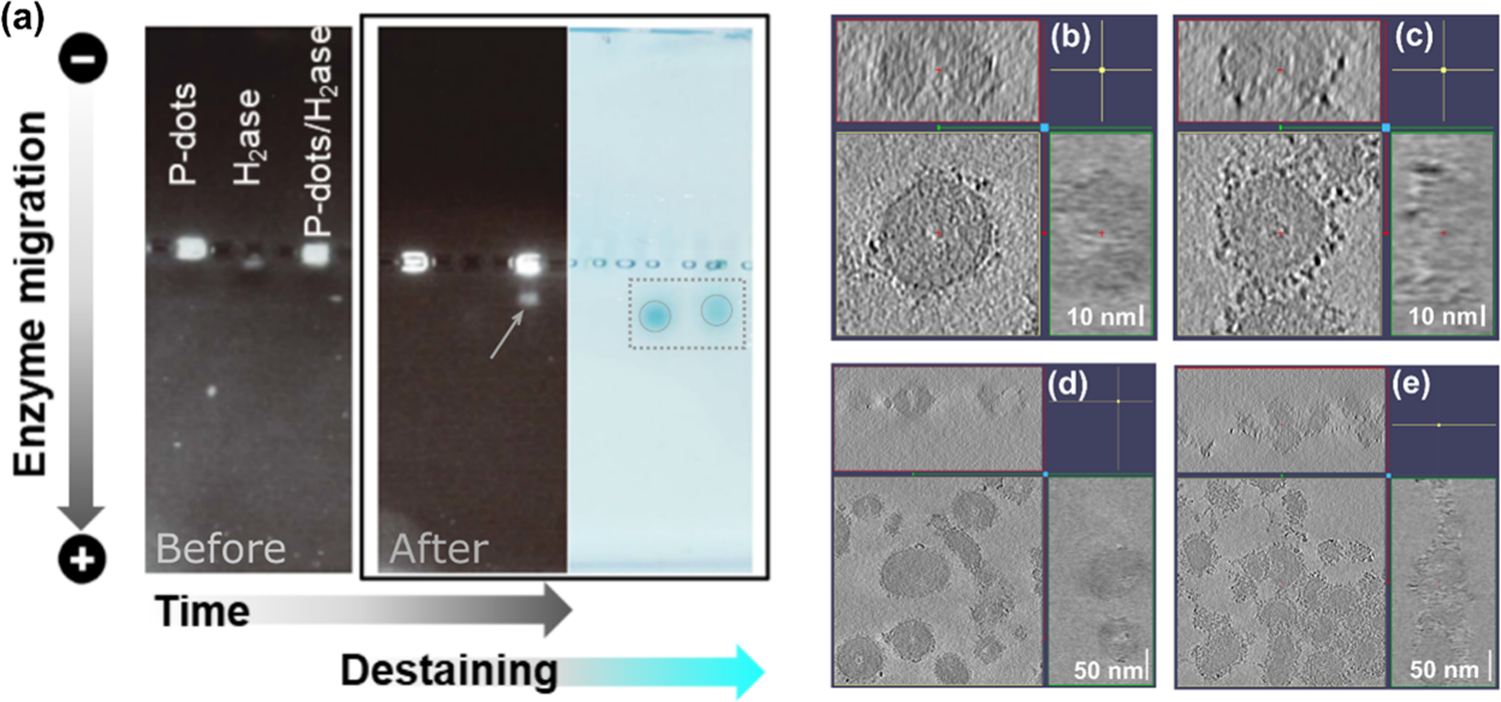
(a) Agarose gel electrophoresis of Pdots, hydrogenase, and Pdots with hydrogenase before and after applied potential (black and white images) and after destaining (light blue image). Note that an excess of Pdots relative to H2ase was employed in these experiments. Orthogonal cross sections of cryo-electron tomograms of P1/P2 Pdots before (b, d) and after incubation with hydrogenase (c, e).

The interaction between the photosensitizer and hydrogenase is very important for interfacial charge transfer, which significantly influences the photocatalytic performance. As determined from surface ξ-potential measurements (Table S1), the surface charge of the binary P1/P2 Pdots is +31 mV. After Pdots were anaerobically incubated with hydrogenase, the measured surface charge changed to ξ = −11 mV (Table S1). The interaction between Pdots and the hydrogenase was at first probed by agarose gel electrophoresis. The gel image (Figure 2a) demonstrated the propagation of Pdots, hydrogenase, and Pdots with hydrogenase through the gel. As shown in Figure 2, in isolation, the Pdots did not migrate upon application of the potential. However, once Pdots were anaerobically incubated with hydrogenase, a fraction of the Pdots appear to copropagate with the enzyme. After destaining, an overlap of bare enzyme bands with these bands observed for the emissive Pdots suggested that the Pdots and the enzyme had migrated through the agarose gel together. As shown in Figure 2a, hydrogenase itself was not emissive. The localization of the hydrogenase band at the same position as the Pdots band indicated that there was an attraction between the binary Pdots and the hydrogenase enzyme. Still, a significant fraction of the Pdots evidently remained immobile also in the presence of the hydrogenase. This was tentatively attributed to the Pdots that did not contain adsorbed hydrogenase and remain stationary upon application of the potential.

Intermolecular interactions between Pdots and the enzyme can be driven by various noncovalent forces, e.g., electrostatic, van der Waals, and hydrophobic effects.^59^ Thus, surface groups, surface charge, and overall morphology are important factors for self-assembly behavior. Bearing a positively charged pocket on the surface near the active site of the enzyme, FeFe-H_2_ase, is known to supply a docking site for small photosensitizers (2–3 nm) with a negative surface charge (e.g., carbon dots,^12^ CdTe nanocrystals^14^). Opposite charge attraction was observed by Reisner et al. for nanoparticles larger than the docking site (*d* = 2.5 nm).^11^ In their case, positively charged ammonium-terminated carbon dots (6.4 ± 1.2 nm) outperformed negatively charged dots because of more intimate interaction and efficient interfacial electron transfer to a [NiFeSe]-hydrogenase.^11^ Taking into account that the average size of positively charged binary Pdots within this study is ∼60 nm, we hypothesized that attraction to the enzyme occurs predominantly via the negative surface charges on the enzyme. However, the role of the hydrophobic attraction via the alkyl side chains of the Pdots, which in turn promotes close-packing with the enzyme, should not be excluded.^60^

The morphology of the binary P1/P2 Pdots was further characterized by cryo-transmission electron microscopy (Cryo-TEM). Cryo-TEM can image the Pdots and enzymes in an environment close to their native state with minimal perturbations. Cryo-TEM imaging shows that binary P1/P2 Pdots are hollow (Figure S7a). This morphology is most likely due to utilization of an amphiphilic block copolymer (e.g., P2 with grafted poly(*N*,*N*-dimethylamino ethyl methacrylate units) during self-assembly preparation.^61^ Cryo-TEM does not allow to distinguish between hollow particles with a ring torus shape or a spherical cap shape architecture. Thus, the 3D morphology of Pdots and control over enzyme orientation on the Pdots surface were investigated using cryo-electron tomography (Cryo-ET) measurements. As shown in Figure 2b,d and Movies (SI), Pdots have a ringed toroid shape (like a donut). The average inner ring diameter is about 10 nm, which is sufficiently large to permit the attachment of enzyme moieties (43 Å × 49 Å × 75 Å) inside the cavity of the Pdots. After incubation of Pdots with hydrogenase, we observed that the hydrogenase units were located with Pdots (Figure 2c), confirming their close interaction as earlier evidenced by gel electrophoresis (Figure 2a). Furthermore, Cryo-ET measurements revealed that H_2_ases were resting on the surface of Pdots (Figure 2e and movies in the SI). This minimizes the electron diffusion distance as both parts, the photosensitizer and the catalyst, are effectively fused.

### Evaluation of the Feasibility of Charge Separation

**Figure 3:**
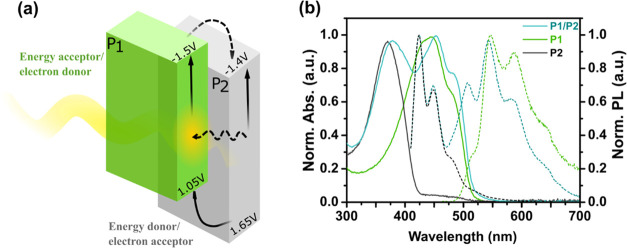
(a) Proposed energy and charge transfer pathways for P1/P2 Pdots. (b) Steady-state UV–vis (solid lines) and photoluminescence spectra (dotted lines) for aqueous solutions of P1 Pdots (green), P2 Pdots (dark gray), and P1/P2 Pdots (blue).

Before using Pdots to drive electrons toward hydrogenase for hydrogen production, the energy potentials of the polymers were investigated. The donor/acceptor (D/A) architecture of heterojunction P1/P2 Pdots is beneficial for overcoming the problem of initially poor charge separation, which is characteristic of organic photocatalysts.^62,63^ Unavoidable losses of free energy can be minimized by selecting D–A structures that operate under driving forces close to zero during stepwise electron transfer.^64,65^ The polymers selected for Pdots formation in this work have type II energy level offset (Figure 3a) with the reduction potential of P1 (*E*_P1/P1^–^_ = −1.5 V vs NHE) being more negative than that of P2 (*E*_P2/P2^–^_ = −1.4 V vs NHE), as estimated from cyclic voltammetry (Figure S8a,c). In this binary composite, P1 acts as the electron-donor polymer and P2 is the electron-acceptor polymer. Under a driving force of 0.1 V, the electrons can accumulate on P2 for the following proton reduction reaction. To gain insights into the feasibility of excited-state hole injection from the polymer to the sacrificial electron donor and between P1 and P2, we estimated values of the excited-state potentials (*E*_P1^–^/P1*_, *E*_P2^–^/P2*_) by subtraction of *E*_0–0_ from the reduction potential of the polymer in the ground state (eqs 1 and 2). The potentials of the polymer in the excited state (*E*_P1^–^/P1*_ = 1.05 V vs NHE, *E*_P2^–^/P2*_ = 1.65 V vs NHE) are found to be sufficiently oxidizing to produce reduced Pdots by the sacrificial electron donor (e.g., triethanolamine, TEOA, 0.82 V vs NHE).^66^ Moreover, it is thermodynamically feasible to have hole injection from P2 to P1 upon light illumination.

### Optical Properties of the Pdots

Pdots have promising light-harvesting and charge transfer capabilities that can be further utilized for efficient hydrogen evolution by the biohybrid assembly.^22−28^ To clarify the beneficial role of the D/A binary architecture, we have prepared Pdots composed of a single polymer, either P1 or P2, to compare their photophysical properties. The absorption spectra of binary P1/P2 Pdots as well as nanoparticles made of a single polymer (P1 or P2) are presented in Figure 3b. The emission spectrum of P1/P2 Pdots (λ_exc._ = 400 nm) is a composition of the emission spectra of P2 and P1 with peaks centered at 420 and 540 nm and originates from P2 and P1 polymers, respectively (Figures 3b and S9). The photoemission quantum yields (QYs) for the as-prepared Pdots were determined using Coumarin 343 in ethanol as a reference: QY_P1_ = 5.5%, QY_P2_ = 49.8%, and QY_P1/P2_ = 15.5%.^52,53^

**Figure 4:**
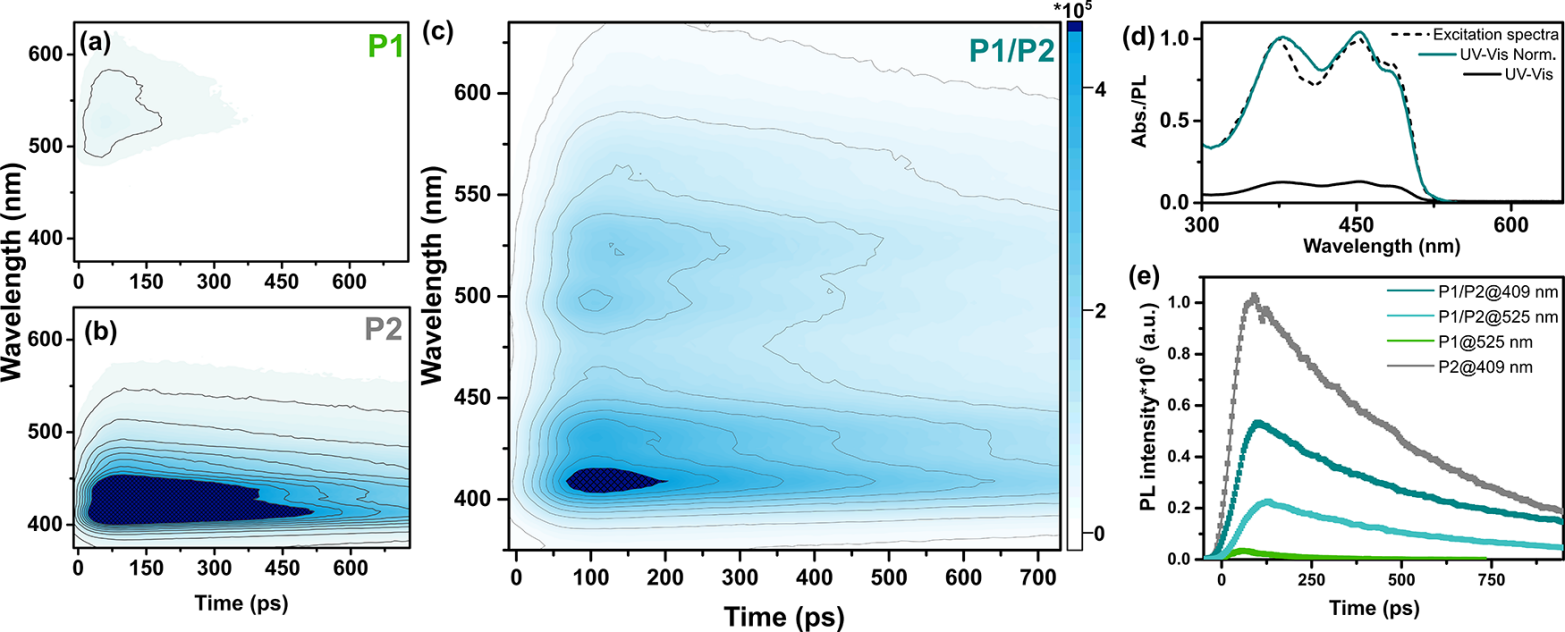
Time-resolved fluorescence spectral decay profiles of P1 Pdots (a), P2 Pdots (b), and binary P1/P2 Pdots (c) measured with a streak camera upon excitation at 370 nm. The scale bar on the left of (c) represents intensity in the corresponding 2D graphs (a–c). (d) Steady-state UV–vis (black) and normalized steady-state UV–vis (cyan) of binary P1/P2 Pdots. The fluorescence excitation spectrum recorded with emission at 680 nm (black dotted). (e) Kinetic traces extracted at 525 nm for P1 Pdots (green) and P1/P2 Pdots (light cyan) and at 409 nm for P2 Pdots (gray) and P1/P2 Pdots (dark cyan).

The characterization of the initial relaxation processes within the excited binary P1/P2 Pdots was performed with a streak camera that allows simultaneous resolution of the emission signals in spectral and temporal domains. Blending of P1 and P2 within the same Pdots (Figure 4c) largely enhanced the emission intensity at 525 nm, which was initially low for single-polymer P1 Pdots (Figure 4a), while suppressing the emission signal at 409 nm (Figure 4b), which was consistent with steady-state fluorescence data (Figure S9). In view of the fact that emission of P2 vastly overlapped with the absorption of P1, energy transfer from P2 to P1 is a plausible relaxation pathway in addition to electron or hole transfer, vide infra. The energy transfer process was further supported by the significant overlap of the binary P1/P2 Pdots absorption with the excitation spectrum (λ_em._ = 680 nm, Figure 4d). Fluorescence kinetic traces were fitted with a sum of exponentials that were convoluted with the instrument response function (IRF; Figure 4e and Table S2; for details, see the SI). Notably, the energy transfer was only observed for binary P1/P2 Pdots, where P1 and P2 are blended in the same particle. Mixing of P1 and P2 Pdots did not result in fluorescence intensity redistribution (Figure S10), highlighting the influence of the distance between interacting polymers for efficient Förster resonance energy transfer (FRET).^67^

Based on the energy levels of the individual polymers in the ground state (Figure 3a), charge separation within the binary P1/P2 Pdots via electron or hole transfer from P1 to P2 is also thermodynamically feasible in parallel to the involved energy transfer previously discussed. To test the possibility of excitons breaking into free charges, i.e., where distinct oxidized and reduced species exist, we performed femtosecond transient absorption spectroscopy (TAS) measurements (Figures S11 and S13). Spectroelectrochemistry was used to support peak assignments (Figure S12). Immediately after excitation (λ_exc._ = 398 nm), a pronounced positive peak centered at ∼700 nm was observed for P1/P2 Pdots with a rise time of 320 fs. By comparison with the absorption spectra for oxidized/reduced polymers obtained by spectroelectrochemistry (Figure S12), we assigned the spectral signature centered at 700 nm to the oxidized P1 polymer (P1^+^).^68,69^ A similar band of oxidized P1^+^ arising from charge separation was reported earlier by Yonezawa et al. for bulk heterojunction F8T2:PC_70_BM blend films.^70,71^ The analysis of TAS data recorded upon excitation at 398 and 490 nm revealed that subpicosecond charge separation occurs predominantly via hole transfer from P2 to P1 (details are given in the SI), while the energy transfer between P2 and P1 polymers occurs via the FRET mechanism on the picosecond timescale.^72^ The recombination between P2^–^ and P1^+^ occurs on the late ps–ns timescale, suggesting that an additional electron acceptor unit might be necessary to remove the electron from P2^–^ and to promote catalysis that typically occurs on the μs–s timescale.

### Photoinduced Electron Transfer from Pdots to the Redox Mediator and the Enzyme

To further our mechanistic understanding, we studied not only the electron flow within binary P1/P2 Pdots but also the ability of synthesized polymer dots to transfer photogenerated charges to the catalyst via a redox mediator. The redox mediator, MV^2+^ with a reduction potential of −0.43 V vs NHE at pH 7,^73^ was added to solutions containing P1/P2 Pdots. The function of the redox mediator was to: (a) suppress the charge recombination between P1 and P2 that occurs on the ns timescale and (b) protect hydrogenase against deactivation at highly reducing potentials.^6,74,75^ Competition between deactivation pathways of the photoexcited state was investigated by steady-state fluorescence quenching experiments. Changes in fluorescence quenching of binary Pdots were tracked as a function of increasing MV^2+^ concentrations. Gradual addition of MV^2+^ resulted in efficient excited-state quenching by electron transfer from binary P1/P2 Pdots to MV^2+^ (λ_exc._ = 400 nm, Figure S15).

The electron transfer properties of Pdots under photocatalytic conditions (white LED, 50 mW·cm^–2^, 420–750 nm) were evaluated in the presence of MV^2+^ (5 mM) and sacrificial electron donors (TEOA or EDTA). Reduction of MV^2+^ resulted in formation of the distinct absorption features of the reduced methyl viologen radical (MV^•+^) at 396 and 603 nm. Binary P1/P2 Pdots showed three to four times higher activity toward MV^2+^ reduction when TEOA was used versus EDTA. This is most likely due to improved regeneration of P1 from P1^+^ by TEOA than by EDTA (*E*_(EDTA)ox_ 0.82 – 1.1 V vs NHE,^66^Figures S16 and S17c). Under photocatalytic conditions, the rate of MV^2+^ reduction was 130 μmol·L^–1^·h^–1^ (Pdots 16 μg·mL^–1^, 10% TEOA, 5 mM MV^2+^; Figure S18 and Table S4). In the presence of TEOA, the amount of MV^•+^ produced was two to four times higher with binary P1/P2 Pdots when compared to the single-polymer P1 Pdots or P2 Pdots (Figure S17, pH = 7), highlighting the beneficial role of initially fast charge separation in the nanoparticles with D/A architecture.

**Figure 5:**
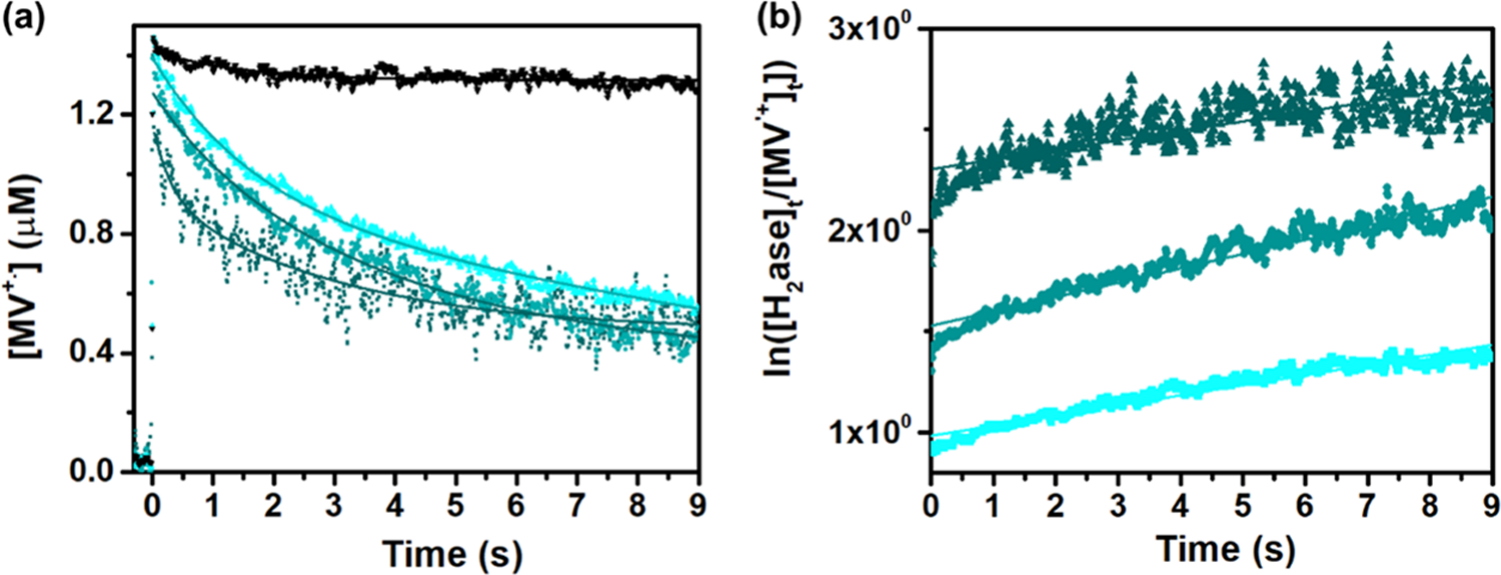
(a) TA kinetic traces recorded at 603 nm for MV^•+^ in reaction mixtures (Pdots 16 μg·mL^–2^, MV^2+^ 5 mM, TEOA 20%, pH 7) without (black curve) and with hydrogenase (8–22.5 μM, cyan blue lines). (b) The fitting result of the 603 nm TA signal for the MV^•+^ population in the presence of hydrogenase applies a model of second-order decay.

To extract the rate constant for the electron transfer to the hydrogenase enzyme, ns-transient spectroscopy studies were performed.^74,75^ The amplitude of the positive TA signal at 603 nm was used to quantify the amount of the generated MV^•+^ (ε_603_ = 13 700 M^–1^cm^–1^). Under the present experimental conditions (Pdots 16 μg·mL^–1^, 5 mM MV^2+^, 20% vol TEOA), around 1.5 μM of MV^•+^ was generated per laser flash upon excitation at 355 nm. In Figure 5, the TA kinetic traces of MV^•+^ population recorded at 603 nm are presented for the reaction mixtures without (black line) and with the enzyme (cyan blue lines). Gradual addition of the hydrogenase enzyme from 8 to 22.5 μM (activity 325 μmol_H2_·mg_H2ase_^–1^·min^–1^) resulted in efficient decay of MV^•+^ population due to an additional electron transfer pathway to the catalyst.^74,75^ Monoexponential fitting was not satisfactory with the kinetic traces in Figure 5 (cyan blue lines), while the kinetic traces were very well fitted with the second-order decay fit function. MV^•+^ population is long-lived; therefore, its decay over time is not considered for the systems in the presence of hydrogenase. The resulting rate constant of electron transfer to hydrogenase was determined to be *k*_1_ = 5.2 × 10^4^ M^–1^ s^–1^. The catalytic cycle of the [FeFe]-hydrogenase enzyme involving the intermediate states was earlier studied in detail by Dyer et al. using laser-induced jump and time-resolved infrared spectroscopy.^74,75^
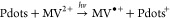
4
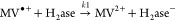
5
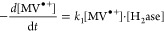
6
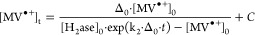
7where Δ_0_ = [H_2_ase]_0_ – [MV^•+^]_0_

### Photocatalytic Hydrogen Evolution

The photocatalytic activity of the Pdots:H_2_ase system was evaluated under solar light simulating conditions (LED, 50 mW·cm^–2^, 420–750 nm) in the presence TEOA or EDTA (pH 7) as sacrificial electron donors. Substitution of EDTA by TEOA gave a fourfold increase of produced hydrogen from 69 ± 2 mmol_H2_·g_H2ase_^–1^ to 286 ± 19 mmol_H2_·g_H2ase_^–1^ (Figure S19, hydrogen evolution data are presented per gram and/or per mole of the catalyst). Blank experiments excluding one of the key components, e.g., the catalyst, light harvester, or redox mediator, in addition to the studies performed in dark showed negligible hydrogen production (Figure S19). The photocatalytic conditions were optimized by varying the concentration of the binary P1/P2 Pdots photosensitizer and resulted in enhanced H_2_ formation (360 mmol_H2_·g_H2ase_^–1^, Figure S20a). The rate of hydrogen production reached a plateau at 10 μg·mL^–1^ Pdots (Figure S20b). When the concentration of Pdots exceeded 30 μg·mL^–1^, the rate declined most likely due to increased light scattering events. A gradual increase in the catalyst concentration facilitated the reaction, reaching the highest rate of hydrogen evolution (43 ± 1 μmol·L^–1^·h^–1^) in the presence of 158 pmol of hydrogenase (Figure S20c, 10% vol TEOA, reaction volume of 2 mL). Further addition of excess enzyme inhibited the rate growth, showing saturation effects or possibly inefficient electron transfer to the enzyme that was no longer attached with the Pdots. Photocatalytic experiments performed in various pH ranges using ascorbic acid (pH = 4.2) or triethanolamine (pH = 6.2, 7, 8, 9) as sacrificial electron donors revealed that the yield of hydrogen production matched well with the activity of the hydrogenase enzyme at the corresponding pH range (Figure S21).^76,77^

**Figure 6:**
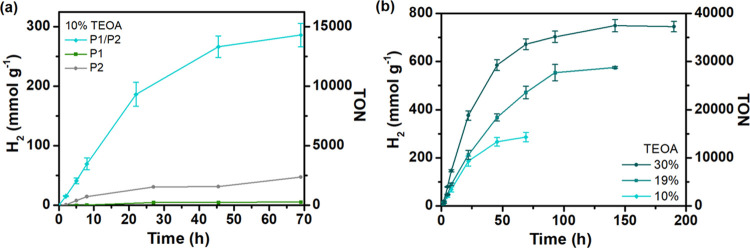
(a) Photocatalytic data for Pdots (16 μg·mL^–1^) based on single polymers P1 (green line) and P2 (gray line) and for binary P1/P2 Pdots (blue line) at pH 7 in the presence of TEOA (10% vol, pH 7), MV^2+^ (5 mM), and hydrogenase (158 pmol) initiated by LED irradiation (50 mW·cm^–2^, 420–750 nm). (b) Photocatalytic data for P1/P2 Pdots (16 μg·mL^–1^), MV^2+^ (5 mM), and hydrogenase (158 pmol) in the presence of various amounts of TEOA (10, 19, 30% vol, pH 7 adjusted with HCl).

In the absence of the D/A structure, neat single-component P2 Pdots with higher surface area produced hydrogen more efficiently compared to the corresponding P1 Pdot system. This is most likely due to shorter distances of free charge carriers’ movement toward the surface of the smaller Pdots or the catalytic active sites provided by the P2 particles (average size ∼20 nm vs P1: ∼80 nm). However, both Pdots composed of a single polymer, either P1 or P2, resulted in 10 (for P2) to 50 (for P1) times lower photogenerated hydrogen yields than for the binary P1/P2 Pdots (Figure 6a). The results suggested that the binary D/A architecture was indeed beneficial for achieving high photocatalytic efficiency in the Pdots–H_2_ase hybrid photocatalytic system. The resulting external quantum efficiencies (EQEs) of 1.1 and 0.3% were obtained during the first 5 h of illumination at 405 and 420 nm, respectively.

The stability and performance of the biohybrid assembly under photocatalytic conditions were also investigated in the presence of various quantities of the sacrificial electron donor (TEOA). Increasing amounts of TEOA from 10 to 30 vol % resulted in a threefold enhancement in the rate of hydrogen evolution (Figure 6b). The biohybrid system was stable up to 100–150 h, reaching 750 mmol_H2_·g_H2ase_^–1^ of photogenerated hydrogen in the presence of 30 vol % TEOA, with a TON of 37 500 ± 1290. The initial activity of the Pdots–hydrogenase hybrid system was 88 460 μmol_H2_·g_H2ase_^–1^·h^–1^ (19% vol TEOA) when 35 pmol of hydrogenase was used (Figure S19b).

In most cases, after a few days of continuous illumination, the hydrogen accumulation plateaued, and freshly injected Pdots could not reinitiate the H_2_ formation process (10% vol TEOA, Figure S22). However, when extra quantities of fresh hydrogenase were injected (158 pmol), photocatalytic proton reduction could be easily restarted (Figure S22), resulting in generation of additional 140 mmol_H2_·g_H2ase_^–1^ hydrogen. These results suggested that the stability of hydrogenase was an issue during photocatalysis. Degradation of the hydrogenase enzyme after prolonged photoirradiation has been observed by several groups.^74,78−80^ In agreement with other reports, we observed the accumulation of a CO-inhibited H-cluster (Hox-CO), as a result of the release of the intrinsic carbonyl ligands from degraded H-clusters that are recaptured by intact H-clusters (Figure S23). The light sensitivity of hydrogenase with and without Pdots was studied by measuring its activity after prolonged illumination. At first, samples containing hydrogenase, MV^2+^, and TEOA (20%, pH 7) with and without Pdots were illuminated for 25 h. Then, the headspace was purged with Ar, and the activity of the remaining hydrogenase was tested by adding fresh MV^2+^ and sodium dithionite. The activity of hydrogenase without Pdots measured with chemical reduction from sodium dithionite was 8439 μmol_H2_·g_H2ase_^–1^·min^–1^, while with Pdots, it was 69200 μmol_H2_·g_H2ase_^–1^·min^–1^. These results suggest that strongly absorbing Pdots protect enzymes against light damage. Moreover, the combination of Pdots with catalase results in the protection of hydrogenase against damage up to 0.3% O_2_ in the headspace (Figure S24b in the SI).^81,82^ In this case, Pdots and MV^2+^ probably reduce molecular oxygen into H_2_O_2_ (Figure S24a),^83^ while the catalase efficiently dismutates produced H_2_O_2_ to half a molecule of O_2_ and water as a side product (Section IV in the SI).

**Figure 7:**
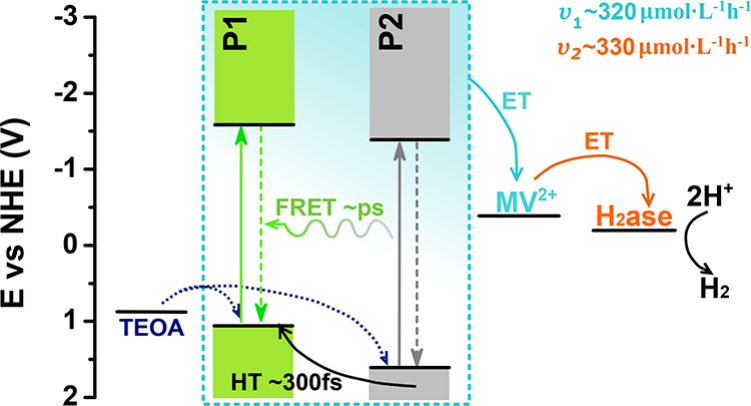
Energy diagram summarizing the photophysical processes involved with biohybrid assembly during photocatalysis. The cyan highlighted portion represents processes of photogeneration and charge separation within heterojunction Pdots (ET—electron transfer, HT—hole transfer). Curved arrows represent charge transfer processes. Rates for MV^•+^ formation (υ_1_) and reduced hydrogenase formation (υ_2_) are reported for the system with 30 vol % TEOA.

Different electron transfer and energy transfer events that take place in the photocatalytic system are summarized in Figure 7. Photoexcitation of binary P1/P2 Pdots results in charge separation on the subpicosecond timescale, while energy transfer occurs on a picosecond timescale. Irradiation of solutions containing binary P1/P2 Pdots in the presence of MV^2+^ and TEOA produced MV^•+^ (υ_1_). The binary P1/P2 Pdots produced significantly more MV^•+^ than P1 or P2 alone (Figure S15), which points to synergistic energy transfer and charge separation in P1 and P2 that enhances MV^•+^ production. Finally, electrons are subsequently transferred to hydrogenase (υ_2_), while the sacrificial electron donor TEOA fills the hole(s) on P1 and P2 to regenerate the Pdots.

To obtain more efficient biohybrid assemblies, the factors limiting conversion efficiency need to be identified. Control photochemical experiments with binary P1/P2 Pdots, MV^2+^, and increasing amounts of TEOA in the absence of hydrogenase showed that the rate of MV^•+^ formation was enhanced (Figure S18 and Table S4) from 130 μmol·L^–1^·h^–1^ (10 vol %, 0.67 M TEOA) to 320 μmol·L^–1^·h^–1^ (30 vol %, 2 M TEOA). In the second series of control experiments, the concentration of MV^•+^ was increased and no appreciable increase in hydrogen production was observed under photocatalytic conditions (Figure S25). In the third series of control experiments, when the TEOA concentration was increased from 10 to 30% for systems containing single-polymer-based Pdots, the yield of hydrogen evolution was enhanced by 24 times for Pdots based on P1 polymer, while almost no effect was observed for Pdots based on P2 polymer (Figure S26). The three control experiments point to the rate-limiting step in hydrogen production being the regeneration of oxidized P1 polymer in binary P1/P2 Pdots by the electron transfer from TEOA (Figure 7).

The UV–vis spectra of Pdots recorded after 200 h of continuous illumination remained nearly identical to the UV–vis spectrum of the Pdots before the photocatalytic reaction (Figure S27), which demonstrated the superb stability of Pdots on long time scales. The biohybrid assembly remained active under continuous illumination for 100–150 h in contrast to 40–50 h with 30 versus 10 vol % TEOA (Figures 6B and S23). The increased performance of the Pdots–hydrogenase hybrid system during photocatalysis can be explained by more efficient formation of MV^•+^ from more photogenerated electrons in Pdots under higher TEOA concentration (Table S4).

## Conclusions

In summary, we developed and investigated a biohybrid assembly of organic binary P1/P2 polymer dots (Pdots) that functioned as efficient light harvesters providing suitable morphology for self-assembly with H_2_ase. A combination of steady-state and ultrafast transient spectroscopic techniques was applied to provide direct insights into the processes of charge photogeneration, separation, and ultimately electron transfer to the enzyme. The beneficial D/A architecture of the designed binary Pdots resulted in efficient subpicosecond charge separation and “useful” conversion to free charged species (>1.6 ns long-lived charge separated state). The introduction of a heterojunction inside the Pdots leads to 10 to 50 higher hydrogen evolution yields than for the corresponding single-polymer Pdots, making the system of Pdots with hydrogenase among the most efficient polymeric-based photocatalytic systems reported to date. In the presence of TEOA, methyl viologen and hydrogenase, the biohybrid P1/P2 system was stable up to 100–150 h, reaching a photocatalytic performance for hydrogen production up to 88460 μmol_H2_·g_H2ase_^–1^·h^–1^ with a TON of 37 500 ± 1290. Excellent water dispersibility, biocompatibility, and suitable surface groups of the Pdots protected hydrogenase against light damage and facilitated the proximate interaction with hydrogenase, as visualized by agarose gel electrophoresis, Cryo-TEM, and Cryo-ET studies. Moreover, having the photosensitizer and the catalyst in close proximity minimized the electron diffusion distance from the redox mediator. This study represents a new strategy to self-assemble a polymeric light harvester (Pdots) with an enzyme. Pdots are facile to prepare and readily functionalized to enable precise tuning of photophysical properties and energy levels. Using Pdots as a photosensitizer and a scaffold onto which enzymes can be directly attached is a straightforward method that can open the way to a broad range of catalytic reactions using different enzymes.

## Supporting Information Available

Size exclusion chromatogram of Pdots; DLS; ξ-potentials; PXRD; ^1^H NMR spectra; ATR-FTIR; spectroelectrochemistry data; TAS spectra; quenching experiments and additional hydrogen evolution data for Pdots; and movies of Cryo-ET data for Pdots before and after hydrogenase encapsulation (PDF)
movies of Cryo-ET data for Pdots before and after hydrogenase encapsulation; Cryo-ET for mutiple Pdots (MP4)
Cryo-ET for mutiple Pdots with H2ase (MP4)
Cryo-ET for single Pdots (MP4)
Cyro-ET for single Pdots with H2ase (MP4)
