# Supplementary material for: Polymer Dots as Photoactive Membrane Vesicles for [FeFe]-Hydrogenase Self-Assembly and Solar-Driven Hydrogen Evolution
Source: J Am Chem Soc. 2022 Jul 21;144(30):13600–11. doi: 10.1021/jacs.2c03882 (PMC9354254; doi:10.1021/jacs.2c03882)
Supplement: Supplementary file 1 — ja2c03882_si_001.pdf [file ja2c03882_si_001.pdf]

Supporting Information:

Polymer Dots as Photoactive Membrane Vesicles for [FeFe]-hydrogenase Self-assembly and Solar-driven Hydrogen Evolution

Mariia V. Pavliuk,[§] Marco Lorenzi,[†] Dustin R. Morado,[θ] Lars Gedda,[§] Sina Wrede,[§] Sara H. Mejias,[§], Aijie Liu,[§] Moritz Senger,[§] Starla Glover,[§] Katarina Edwards,[§] Gustav Berggren,[*,†] Haining Tian[*,§]

[§]Department of Chemistry - Ångström Laboratory, Physical Chemistry, Uppsala University, 751 20 Uppsala, Sweden
[†]Department of Chemistry - Ångström Laboratory, Molecular Biomimetics, Uppsala University, 751 20 Uppsala, Sweden
[θ] Department of Biochemistry and Biophysics, Science for Life Laboratory, Stockholm University, 171 65 Solna, Sweden

**Table of Content**	**Page**
**I. Preparation of Hydrogenase (expression, purification, reconstitution and maturation)**	S3
**Figure S1.** Size exclusion chromatogram of Pdots (P1/P2).	S4
**Figure S2.** Distribution of the hydrodynamic diameter of Pdots.	S4
**Table S1.** ξ-potentials for P1/P2 binary Pdots before and after incubation with hydrogenase, and zeta potentials for single polymer Pdots P1, P2.	S5
**Figure S3**. UV-Vis spectra and calibration curves for P1 (a) and P2 (b) in THF	S5
**Figure S4.** Powder X-ray diffraction (PXRD) patterns of polymer dots composed of single polymers (either P1 or P2) and PXRD pattern of lyophilized P1/P2 Pdots	S5
**Figure S5.** ^{1}H NMR spectra for P1, P2 and P1/P2.	S6
**Figure S6.** ATR-FTIR spectrum of P1/P2 Pdots	S6
**Figure S7.** Cryo-TEM micrographs of Pdots without (a) and with hydrogenase(b)	S7
**Figure S8.** Cyclic voltammograms of P1 and P2.	S7
**Figure S9.** Steady-state photoluminescence spectra of polymer dots.	S8
**Figure S10.** Steady state PL spectra of P1, P2 Pdots, and P1 Pdots and P2 Pdots mixed together	S8
**II. Relaxation processes within the excited binary P1/P2 Pdots**	S9
**Table S2.** Recombination dynamics extracted from time-resolved streak camera measurements	S9
**Table S3.** Recombination dynamics extracted from TCSPC	S9
**III. Photoinduced charge separation within the binary P1/P2 Pdots**	S9
**Figure S11.** Transient absorption spectra of polymer dots P1, P2, and P1/P2 obtained in a probe interval of 360–710 nm upon 398 nm excitation.	S10
**Figure S12.** Spectroelectrochemistry data collected for reduced and oxidized polymers	S10

**Figure S13.** Transient absorption spectra of polymer dots P1/P2 obtained in a probe interval of 360–710 nm upon 490 nm excitation.	S11
**Figure S14.** TAS decay kinetics of P1, P2 and P1/P2 polymer dots.	S12
**Figure S15.** Fluorescence quenching experiments for binary Pdots with methyl viologen	S13
**Figure S16.** Photoreduction of methyl viologen ($MV^{\bullet+}$) by P1/P2 Pdots in EDTA over time	S13
**Figure S17.** Photoreduction of methyl viologen ($MV^{\bullet+}$) by P1, P2 and P1/P2 Pdots in TEOA	S13
**Figure S18.** Kinetics of methyl viologen photoreduction after illumination of P1/P2 Pdots in presence of different amount of sacrificial electron donor	S14
**Table S4.** Rates for methyl viologen reduction and hydrogen evolution in the presence of different amount of TEOA	S14
**Figure S19.** Hydrogen evolution data for polymer dots with hydrogenase in TEOA and in EDTA, as well as blank experiments that exclude either polymer dots or hydrogenase.	S14
**Figure S20.** Hydrogen evolution data and corresponding rates of H_2 formation in the presence of various quantities of polymer dots or hydrogenase enzyme.	S15
**Figure S21.** Photocatalytic data for binary Pdots in various pH range	S15
**Table S5.** The table comparing the performance of Pdots:H_2ase assembly with the related state-of-the art systems	S16
**Figure S22.** Addition of extra fresh quantities of Pdots or hydrogenase after 48 h of irradiation.	S17
**Figure S23.** ATR-FTIR spectra of reaction mixture during first minutes of LED illumination	S17
**IV. The effect of Pdots on the intrinsic oxygen tolerance of hydrogenase enzyme**	S18
**Figure S24** O_2 reduction studies of P1/P2 Pdots and photocatalysis of P1/P2 Pdots with and w/o catalase in the presence of oxygen	S18
**Figure S25.** Hydrogen evolution data in the presence of various quantities of methyl viologen	S19
**Figure S26.** Photocatalytic data for Pdots based on single polymers P1 and P2 in presence of various amount of TEOA	S19
**Figure S27.** UV-Vis spectra of Pdots before photocatalysis and after photocatalysis during extended illumination.	S19
**References**	S20

I. Preparation of Hydrogenase (expression, purification, reconstitution and maturation).

The hydrogenase, CrHydA1 (M_w 50 kDa) was prepared following literature protocols, and the specific activity was 325 μmol(H_2)·mg^{-1}·(hydrogenase)·min^{-1}.[1,2]

Expression and purification: A BL21(DE3) *E.coli* strain was transformed with a pET11a plasmid carrying a codon-optimized sequence for the expression of *C. reinhardtii* HydA1 in its *apo*- form, cloned in frame to a StrepTagII sequence, and an ampicilline-resistance selection marker. The strain was grown on LB media complemented with 25 mM potassium phosphate buffer, 5 % Glucose and 100 μM ampicilline in open shaking flasks at 37°C until OD_{600}=0.8 – 1; expression was then induced adding 0.5 mM IPTG to the culture and carried on at 16°C for 14-16 h. Cells were harvested via centrifugation, resuspended in lysis buffer (100 mM Tris/HCl pH 8, 200 mM KCl, 25 mM $MgCl_2$ buffer with added lysozyme, DNAse and RNAse) and lysed via sonication. The lysate was then clarified via ultracentrifugation (50000 rpm, 45min, 4°C). The clarified lysate was the loaded on a StrepTrap® column for affinity chromatography purification. 100 mM Tris/HCl pH 8, 200 mM KCl, 5 % glycerol was used as equilibration buffer, and washing buffer. 100 mM Tris/HCl pH 8, 200 mM KCl, 5 % glycerol with the addition of 3mM desthiobiothin was used as elution buffer.

Reconstitution: The purified enzyme was incubated with 20 mM sodium dithionite and 10mM EDTA overnight at 4°C, at the end of which the reaction mix was run through a PD10 desalting column equilibrated with 50 mM Tris/HCl pH 8, 150 mM KCl buffer. The medal-free enzyme obtained was diluted down to 100-150 μM and incubated with 10x molar excess of DTT and 6x molar excess each of L-cysteine and Mohr's Salt; the reaction was then started by adding 1-2% of CsdA. The reconstitution was followed until completion by monitoring the UV/Vis spectrum, then the reaction mix was again cleaned using a PD10 analogously as for the previous step.

Maturation: After semi-enzymatic [4Fe4S] cluster reconstitution, the enzyme was matured using a $Fe_2(adt)(CO)_4(CN)_2^{2-}$ (adt = azadithiolate, $^-SCH_3NHCH_3S^-$) synthetic mimic ($[2Fe]^{adt}$). A mixture containing 100-150 μM reconstituted HydA1, 6x molar excess of $[2Fe]^{adt}$, 2 mM sodium dithionite was prepared in a 100 mM Tris/HCl pH 8, 200 mM KCl buffer; the mixture was incubated for 90 minutes at room temperature and excess mimic and dithionite were removed using a PD10 desalting column, pre-equilibrated with a 100 mM Tris/HCl pH 8, 200 mM KCl buffer.

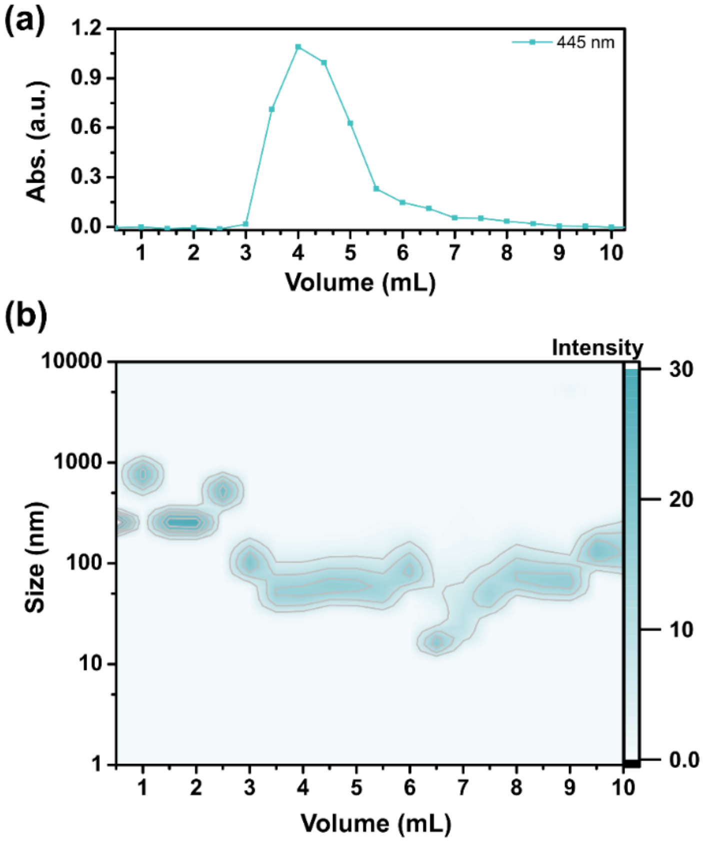

Figure S1. Size exclusion chromatograms of polymer dots (P1/P2) separated by size on a Sepharose CL-4B column eluted with water at a flow rate of 0.5 mL/min. (a) Absorption of Pdots at 445 nm from the collected eluted fractions (0.5 mL) determined by UV-Vis spectroscopy.

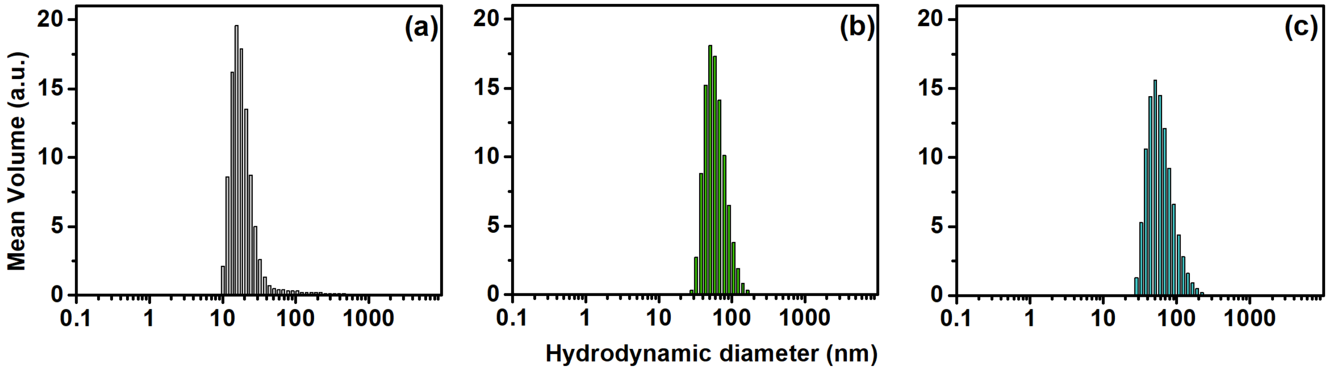

Figure S2. Distribution of the hydrodynamic diameter of Pdots composed of single polymer P2 (a), P1 (b), and mixed Pdots composed of both P1 and P2 (c).

Table S1. ξ-potentials for P1/P2 binary Pdots before and after incubation with hydrogenase, and zeta potentials for single polymer Pdots P1, P2.

Sample	Zeta potentials (ξ, mV)
P1 Pdots	–26
P2 Pdots	30
P1/P2 Pdots	31
P1/P2 Pdots&H_2ase	-11

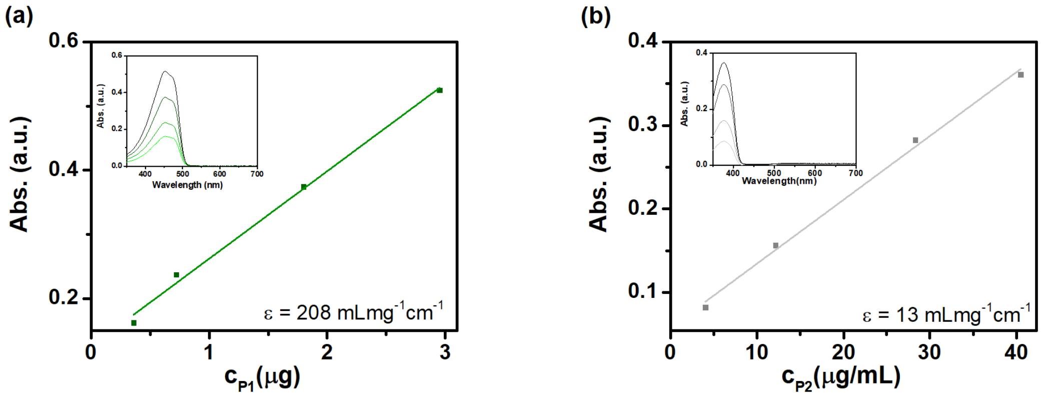

Figure S3. UV-Vis spectra of P1 (a) and P2 (b) in THF in insets, and corresponding calibration curves used for the determination of polymer content in the final mixture with determined extinction coefficients.

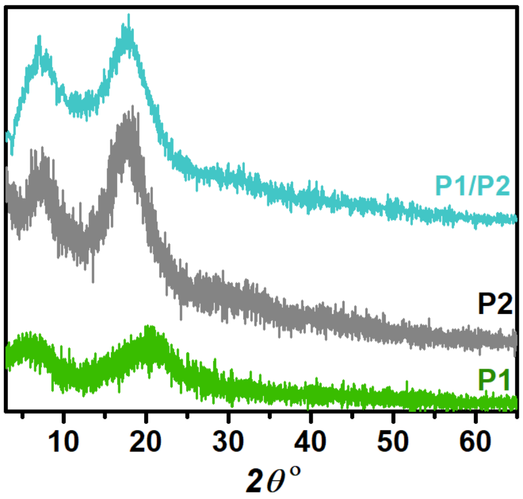

Figure S4. Powder X-ray diffraction (PXRD) patterns of polymer dots composed of single polymers (either P1 or P2) and PXRD pattern of lyophilized P1/P2 Pdots (crystallinity degree 62 %).[3] The diffraction peaks at 2θ of 21° correspond to π-π stacking of P2 backbones; peaks at 6° and 7° corresponding to lamellar packing.

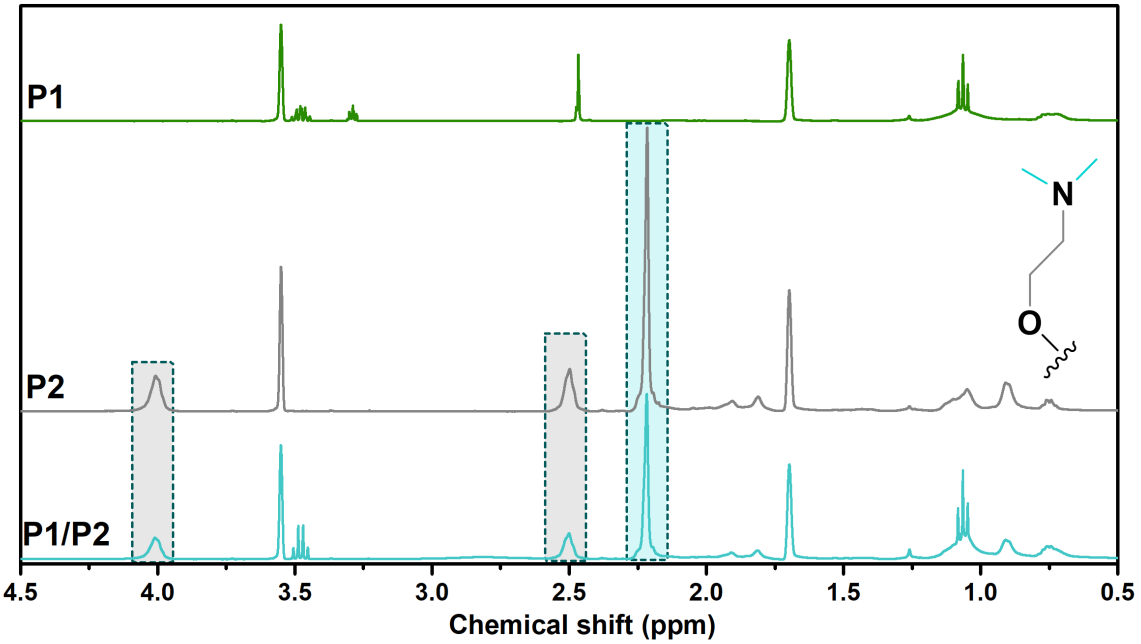

Figure S5. ^{1}H NMR spectra for P1, P2 and P1/P2 in d_8-THF. The signals for ethyl and methyl proton signals are marked with gray and blue boxes, respectively.

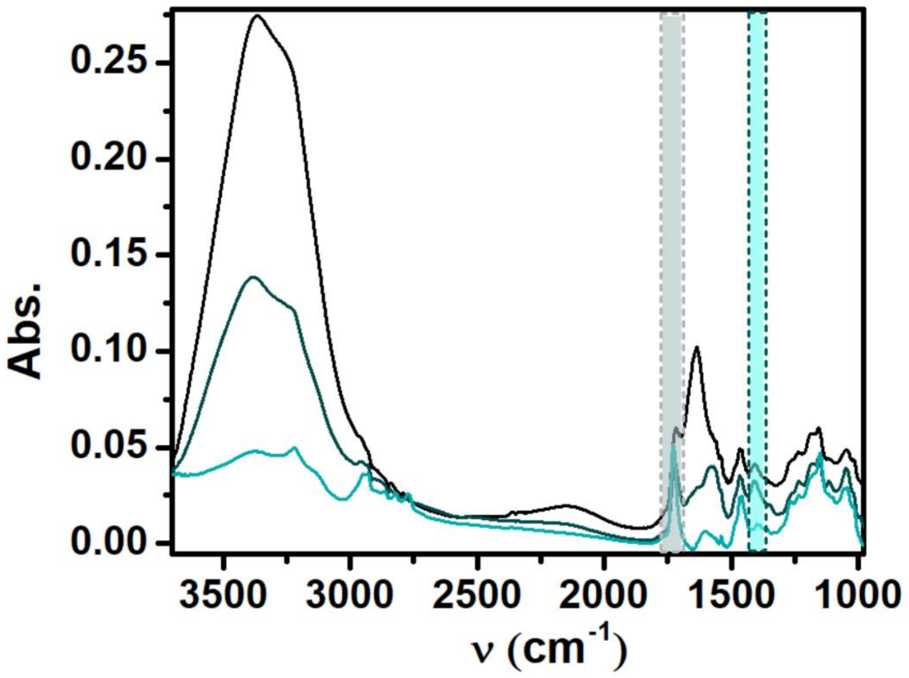

Figure S6. ATR-FTIR spectra of P1/P2 Pdots recorded before (black line), after dehydration (cyan line), and under the stream of "wet" N_2 aerosol (dark cyan line). C=O and C–N vibrational stretches are high-lighted in gray and cyan respectively. The FTIR spectrometer (Vertex70v, Bruker Optik, Germany) that was equipped with a triple-reflection ZnSe/Si crystal ATR cell (BioRadII from Harrick). The infrared spectra were recorded with 80 kHz scanning velocity at a spectral resolution of 2 cm^{-1}.

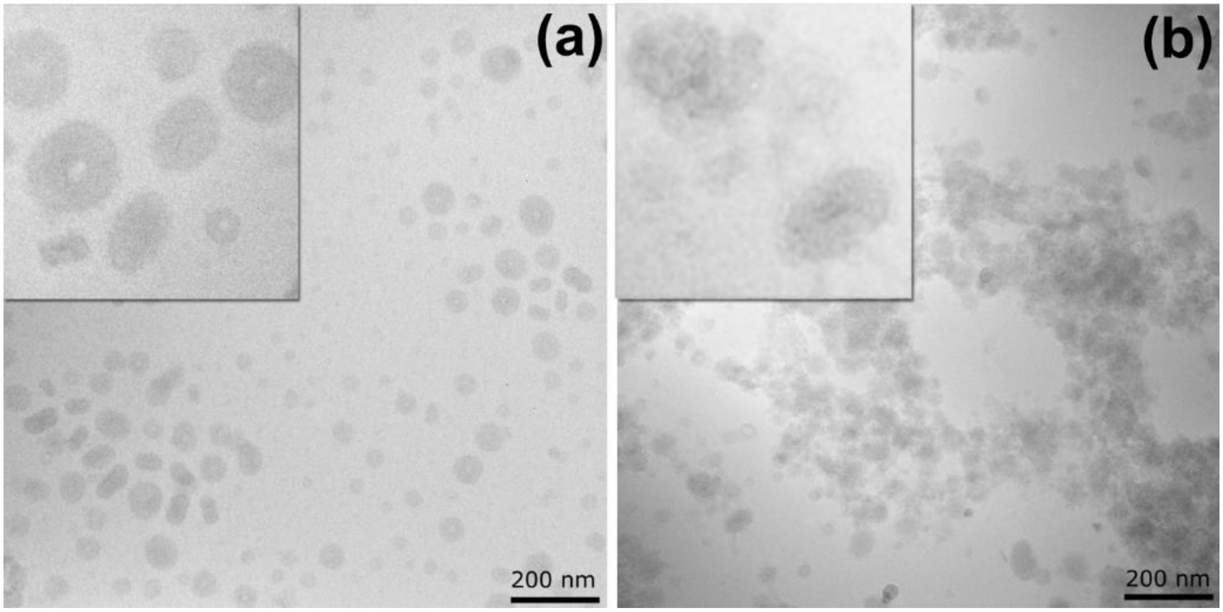

Figure S7. Cryo-TEM micrographs of Pdots without (a) and with hydrogenase(b)

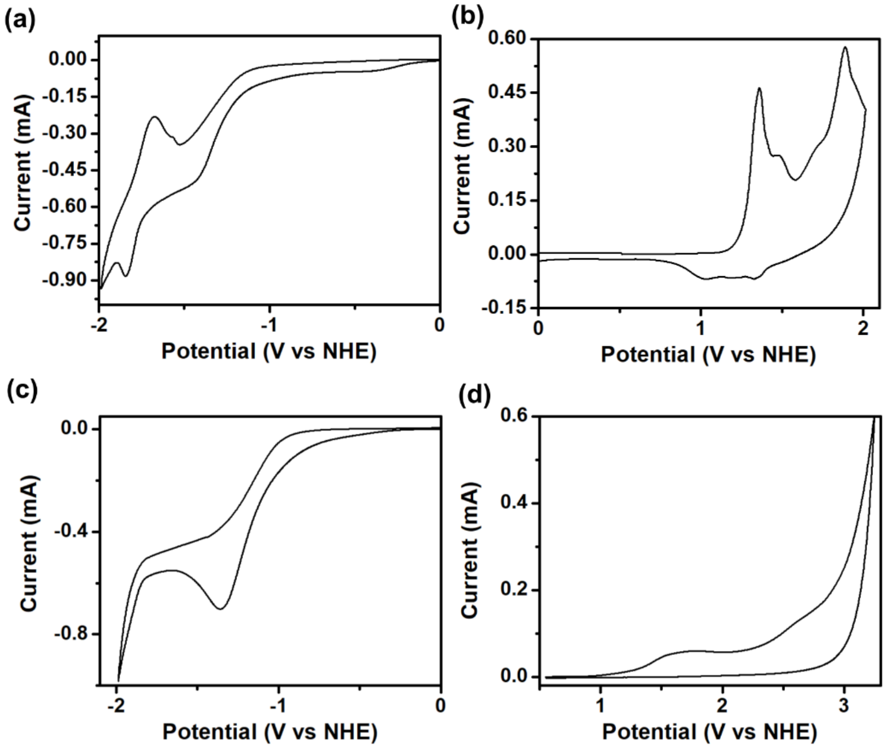

Figure S8. Cyclic voltammograms of polymers P1 (a-b) and P2 (c-d) coated on fluorine-doped tin oxide (FTO) glass measured in acetonitrile with tetrabutylammonium hexafluorophosphate as supporting electrolyte (0.1 M, previously dried at 80 °C in a vacuum oven) at scan rate 50 mV/s. Ferrocene (Fc) was used as internal reference to calibrate the potential of the $Ag/AgNO_3$ electrode to the Normal Hydrogen Electrode (NHE) by adding 0.63 V vs. NHE.[4] Reduction potentials were estimated as E_{peak} potentials.

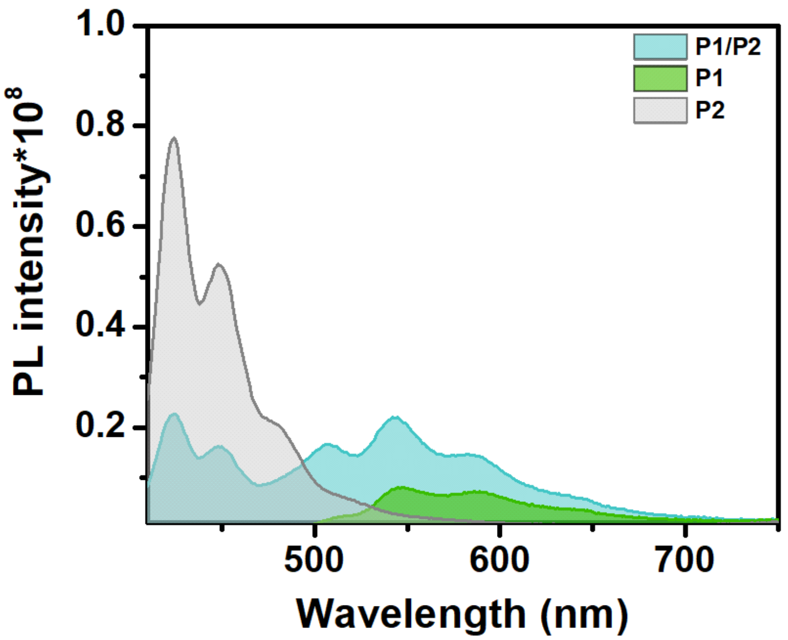

Figure S9. Steady-state PL spectra of polymer dots (P1/P2 – blue, P1 – green, P2-gray) upon excitation at 400 nm.

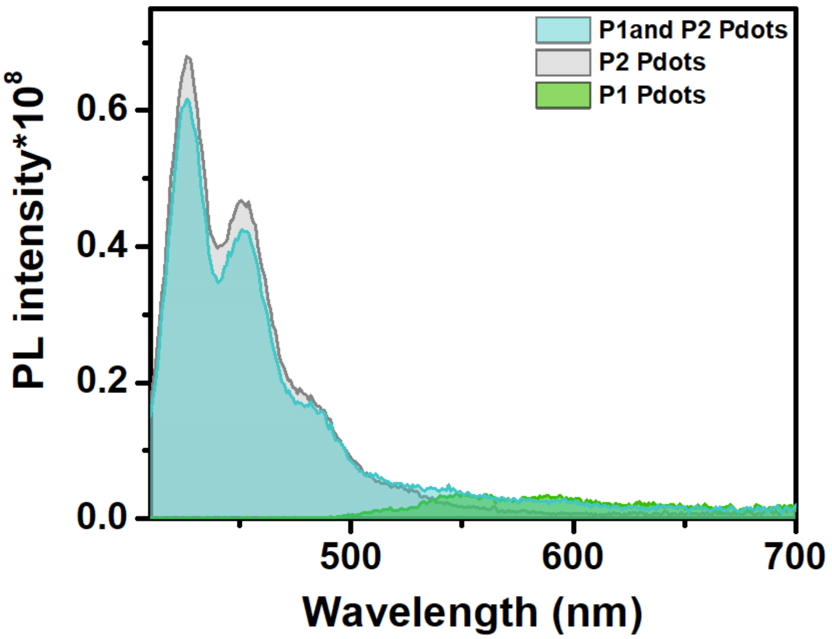

Figure S10. Steady state PL spectra of P1, P2 Pdots, and P1 Pdots and P2 Pdots mixed together upon excitation at 400 nm.

II. Relaxation processes within the excited binary P1/P2 Pdots

Fluorescence kinetic traces were fitted with a sum of exponentials that were convoluted with the instrument response function (IRF, **Figure 4e**, **Table S2**). The origin of components ranging from tens to hundreds of picoseconds observed for P1 (τ_1 ~ 33 ps, τ_2 ~ 157 ps), and for both P2 and P1/P2 Pdots (τ ~ 567 ps and τ ~ 548 ps respectively) could be due to geminate and/or nongeminate recombination of excitons that typically occur on ps-ns timescale.[5,6] Similar relaxation times were reported by Heeger *et al*. for PCDTBT polymer, where they suggested that both exciton formation and migration occurred at much longer timescales than the ultrafast photoinduced electron transfer (~200 fs) in corresponding heterojunction blends.[7] The rise time for the kinetic trace extracted at the emission maximum for single P1 Pdots (525 nm) is around ~56 ps, while it is almost double for the binary P1/P2 Pdots ~102 ps. This delay observed for the heterojunction Pdots is caused by the energy transfer from P2 to P1 within blended Pdots. Due to the narrow probed window of the streak camera, the longer relaxation processes were further analyzed with TCSPC (**Table S3**).

Table S2. Recombination dynamics extracted from time-resolved streak camera measurements

**Sample**	**Lifetimes**		
**@530nm**	τ_1(ps)	τ_2 (ps)	τ_{inf} (ps)
P1-dots	33 (45%)	157(53%)	(2 %)
P1/P2-dots	483 (97%)	-	(3 %)
**@409nm**	τ_1(ps)	τ_2 (ps)	τ_{inf} (ps)
P2-dots	567 (97%)	-	(3 %)
P1/P2-dots	548 (94%)	-	(6%)

Table S3. Recombination dynamics extracted from TCSPC

**Sample**	**Lifetimes**	
**@540nm**	τ_1(ns)	τ_2 (ns)
P1-dots	0.135 (97%)	2.5 (3 %)
P1/P2-dots	0.483 (92.8%)	2.57 (7.2 %)
**@425nm**	τ_1(ns)	τ_2 (ns)
P2-dots	0.63 (72 %)	1.3 (28 %)
P1/P2-dots	0.54 (82 %)	1.3 (18 %)

III. Photoinduced charge separation within the binary P1/P2 Pdots.

The transient absorption spectra for P1 Pdots, P2 Pdots and binary P1/P2 Pdots obtained upon excitation at 398 nm are shown on **Figure S11**, while detailed description of the TAS kinetics is provided in **Figure S14**. At an excitation wavelength of 398 nm the photon absorption efficiency for P1 is 60% in the binary P1/P2 Pdots.

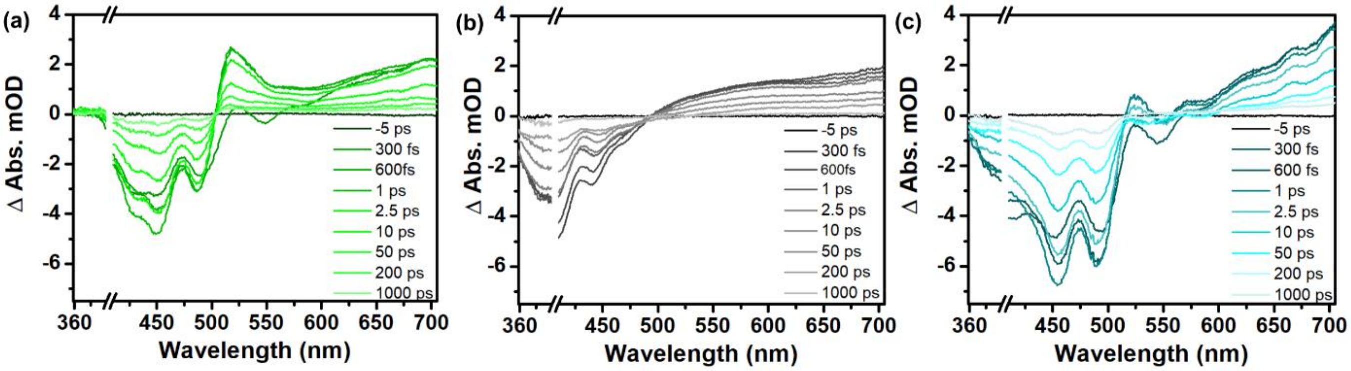

Figure S11. Transient absorption spectra of polymer dots P1 (a), P2 (b), P1/P2 (c) obtained in a probe interval of 360–710 nm upon 398 nm excitation.

Two characteristic excited state features, namely the ground state bleach (GSB) centered at 380 and 450 nm, as well as the stimulated emission (SE) centered at 430 and 550 nm were observed at early time-scales for the P2 and P1 components, respectively. Immediately after excitation a pronounced positive peak centered at ~700 nm was observed for P1/P2 Pdots with a rise time of 320 fs. By comparison with the absorption spectra for oxidized/reduced polymers obtained by spectroelectrochemistry (**Figure S12**), we assigned the spectral signature centered at 700 nm to the oxidized P1 polymer ($P1^+$).[8,9] A similar band of oxidized $P1^+$ arising from charge separation was reported earlier by Yonezawa *et al*. for bulk hetero-junction F8T2:PC_{70}BM blend films.[10,11]

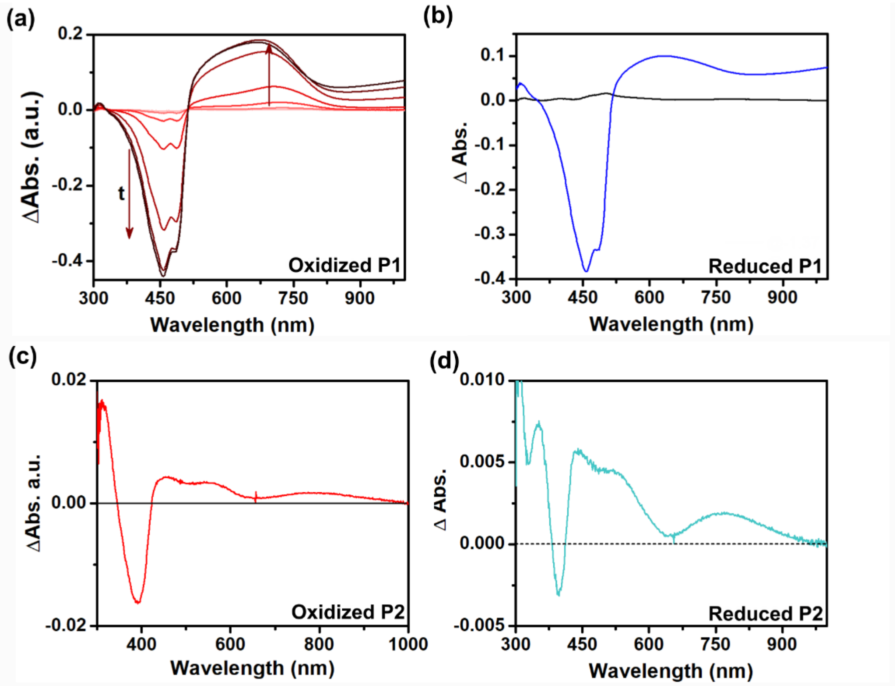

Figure S12. Spectroelectrochemistry data collected for polymers coated on FTO glass as a working electrode in acetonitrile. Corresponding UV-Vis spectra of oxidized P1 polymer (a, collected at 1.4 V vs NHE), reduced P1 polymer (b, collected at -1.5 V(black) and -2 V(blue line) vs NHE), oxidized P2 (c, collected at 1.8 V vs NHE) and reduced P2(d, collected at -1.4 V vs NHE).

The charge transfer within binary P1/P2 Pdots with formation of $P1^{+}$ may occur via direct electron transfer from P1 to P2 or via hole transfer from P2 to P1. As upon excitation at 490 nm (only P1 can be excited), no clear spectral signatures from $P1^{+}$ were observed (**Figure S13**), we conclude that charge separation occurs predominantly via hole transfer from P2 to P1. However, direct electron transfer from P1 to P2 upon excitation at 400 nm should not be completely excluded considering the small estimated driving force of 0.1 V. The sub-picosecond rise time observed for the current system is similar to those observed for other polymer heterojunctions,[12,13] where extensive exciton delocalization occurs without the necessity for its diffusion within the polymer network. Charge transfer between P1 to P2 should result in formation of reduced P2 species ($P2^{-}$). Due to dramatically lower extinction coefficient of P2 polymer (16 times lower than for P1, **Figure S3**) as well as location of its reduced absorption features outside the probed range (below 330 nm and above 700 nm), no clear spectral signatures were found for $P2^{-}$.

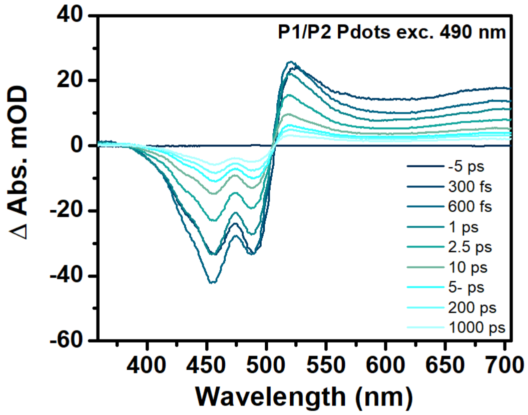

Figure S13. Transient absorption spectra of polymer dots P1/P2 obtained in a probe interval of 360–710 nm upon 490 nm excitation.

Comparison of the kinetic traces extracted at 380 nm (**Figure S14 e,i**) revealed much faster recovery of GSB for binary P1/P2 Pdots than for neat P2 Pdots, resulting in the corresponding lifetimes of 3.3 ps (72%), and 467 ps (25%). Along with recovery of the GSB for P2, GSB for P1 was induced with the rise time of 545 fs, further confirming the earlier results of the involved energy transfer on the early picosecond timescale.

Based on analysis of the steady state and transient results we were able to detail the photoinitiated charge transfer processes involved within binary D/A Pdots. Excitation of the P1/P2 Pdots initiates sub-picosecond charge separation, while the energy transfer between P2 and P1 polymers occurs via FRET mechanism on the picosecond timescale.[5]

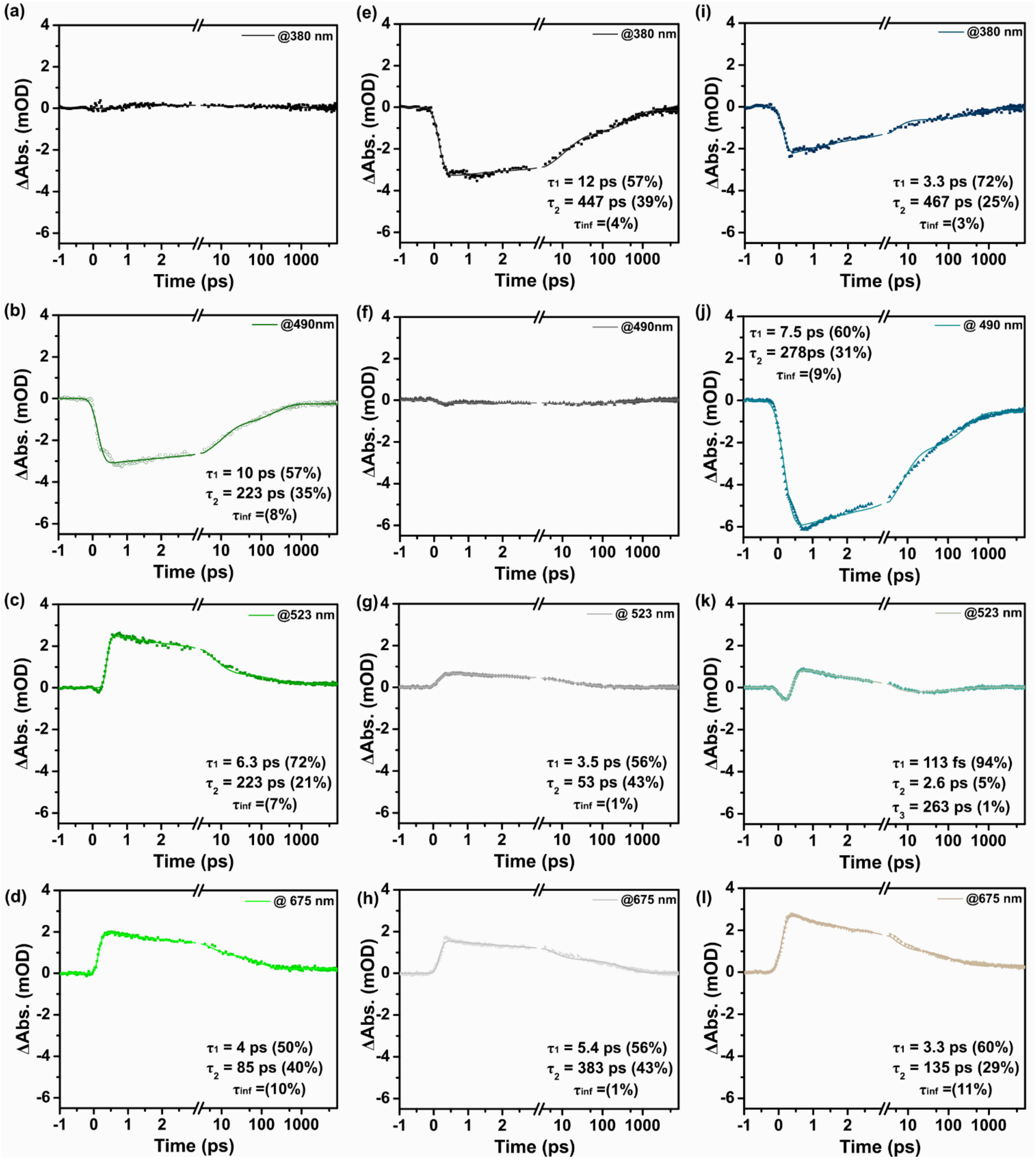

Figure S14. TAS decay kinetics of P1 (a-d), P2 (e-h) and P1/P2 (i-l) polymer dots upon 400 nm excitation extracted at 380 nm, 490 nm, 523 nm and 675 nm respectively. In all transient absorption spectra of polymer dots (Figure 3), the ΔOD spectra consist of negative signals (GSB, SE) in the short-wavelength region and positive features in the long-wavelength region. Ground state bleach bands were centered at 380 and 450 nm for P2 dots and P1 dots respectively, and the corresponding kinetic traces extracted at these wavelengths are presented in Figure S10 (a-b, e-f). The negative signals ascribed to stimulated emission were centered at 430 nm (P2 dots) and 550 nm (P1 dots), and were observed in TA spectra at early timescales. P1 dots has an additional band centered at 520 nm that is assigned to internal charge transfer band of P1 polymer. Addition of P2 in binary Pdots results in suppression of internal charge transfer band most likely due to electron transfer from P1 to P2 in binary Pdots (Figure S10c, and S10k).

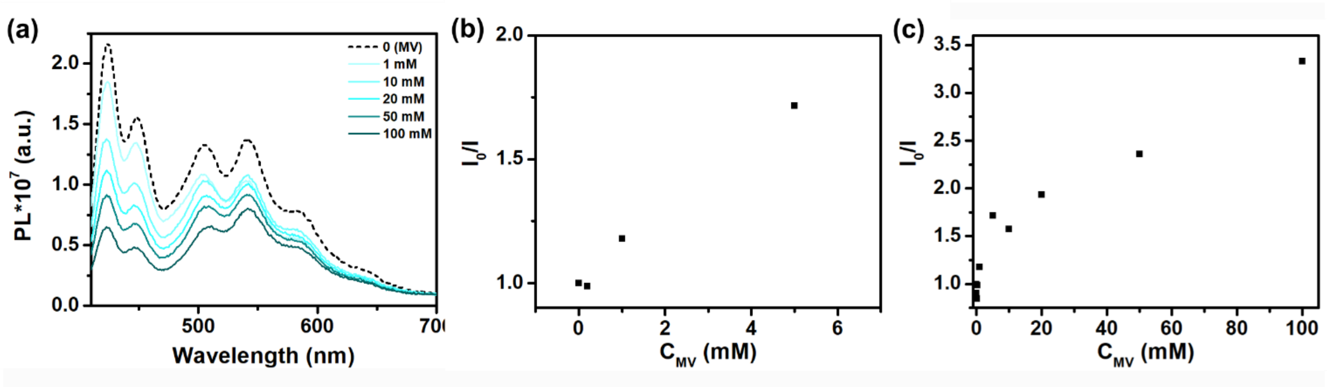

Figure S15. Fluorescence quenching experiments for binary Pdots (absorption is 0.05 at the excitation wavelength, 400 nm) with methyl viologen (a). (b-c) The corresponding Stern-Volmer plots with concentration of quencher (MV^{2+}) from 0.2-5 mM (b) and 0.2-100 mM (c) respectively.

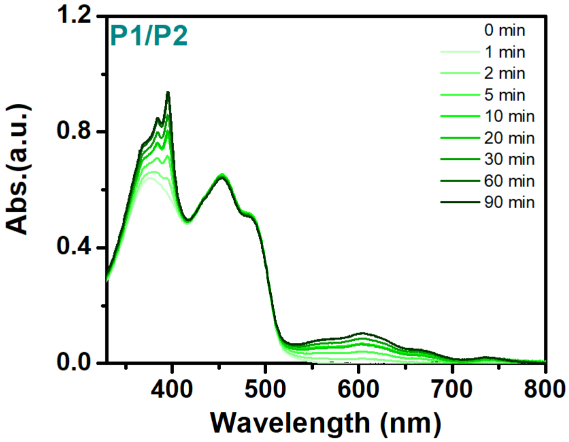

Figure S16. Generation of photoreduced methyl viologen ($MV^{\bullet+}$) after illumination of P1/P2 Pdots in EDTA over time (white LED, 50 mW cm^{-2}, 420−700 nm, EDTA 0.1 M, pH 7).

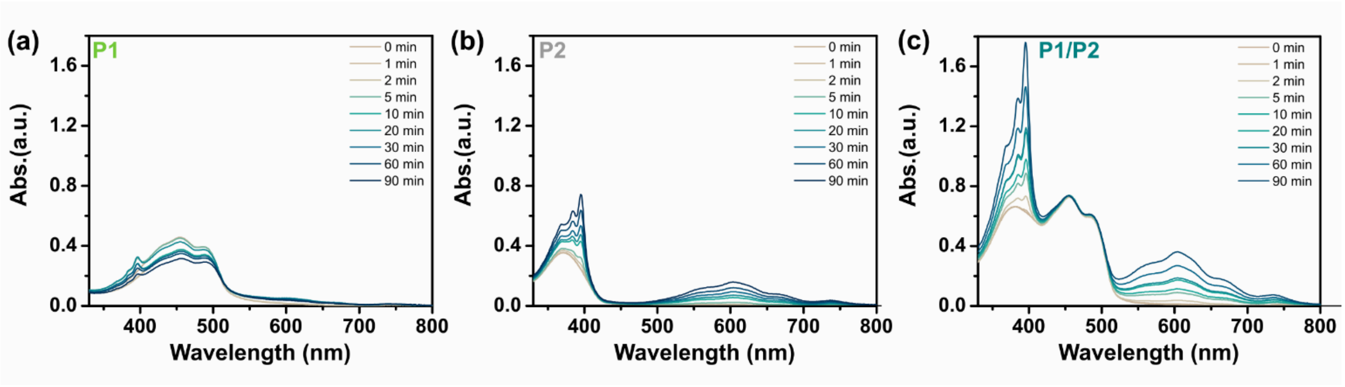

Figure S17. Generation of photoreduced methyl viologen ($MV^{\bullet+}$) after illumination of P1 Pdots(a), P2 Pdots (b), and P1/P2 Pdots (c) over time (white LED, 50 mW cm^{-2}, 420−700 nm, TEOA: 10%0.67 M, pH 7).

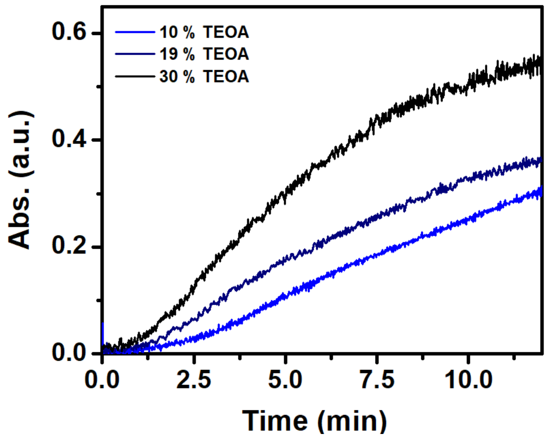

Figure S18. Kinetics of 5 mM methyl viologen photoreduction after illumination of P1/P2 Pdots over time (white LED, 50 mW cm^{-2}, 420−700 nm, TEOA: 10%,0.67 M; 19%, 1.27 M; 30%, 2M; pH 7) monitored by absorption at 605 nm. Rates of reduced methyl viologen formation were calculated by taking the slope from 2.5 to 6 minutes. Rates are reported in Table S3.

Table S4. TOF and rates for reduced methyl viologen formation (**$\upsilon_{Pdot-MV}$**) and rates of reduced hydrogenase formation (**$\upsilon_{MV-H2ase}$**) in the presence of different amount of TEOA.

TEOA (%)	$\upsilon_{Pdot-MV}$ (μmol·L^{-1}h^{-1})	$\upsilon_{MV-H2ase}$ (μmol·L^{-1}h^{-1})	TOF (mol$_{H2}$·mol$_{H2ase}$$^{-1}$h^{-1})
10	130 ± 2	86 ± 4	1088±50
19	200 ± 1	196 ± 8	2480±100
30	320 ± 3	334 ± 12	4225±150

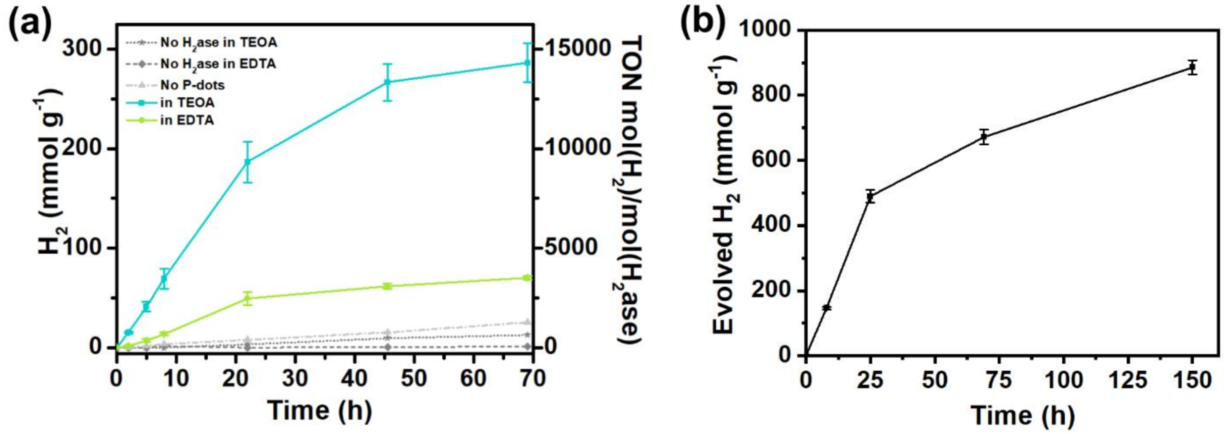

Figure S19. (a) Hydrogen evolution data initiated by LED irradiation (50 mW/cm^2, 420–750 nm) for polymer dots (16 µg/mL) with hydrogenase (79 nM) in TEOA (10 vol%, 0.67 M, light blue line) and in EDTA (0.1M, light green line); as well as blank experiments that exclude either polymer dots (light gray line with triangles) or hydrogenase (in TEOA – dark gray line with stars, in EDTA – dark gray line with rhombi). (b) Hydrogen evolution data for P1/P2 polymer dots (16 µg/mL) with hydrogenase (35 pmol) in TEOA (19 vol%).

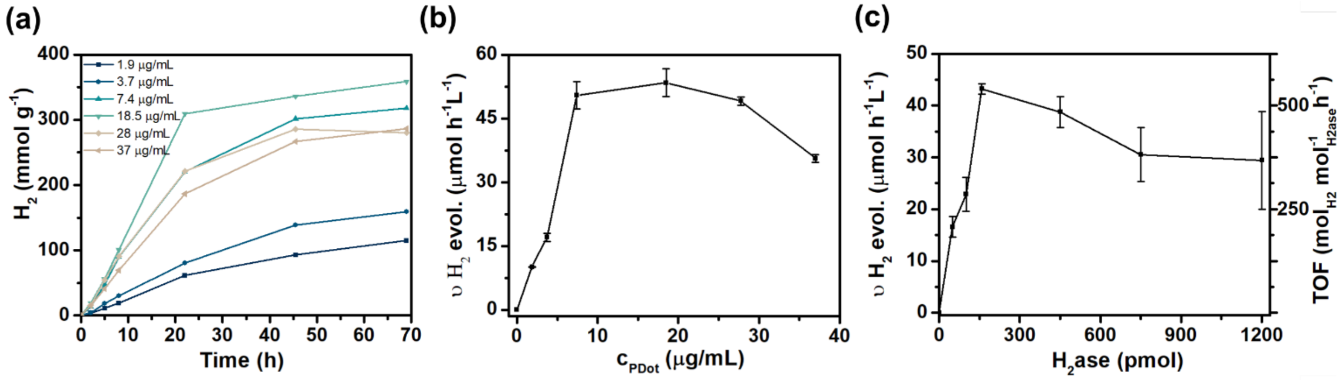

Figure S20. (a) Hydrogen evolution data in the presence of various quantities of polymer dots (2.5–50 µg/ml) with methyl viologen in 10 vol%, 0.67M TEOA. (b-c) Rates of hydrogen evolution in the presence of various quantities of polymer dots (b, 1.9–40 µg/ml) or hydrogenase enzyme (c, 50–1200 pmol) with methyl viologen (5 mM) in TEOA (10 %, 0.67 M).

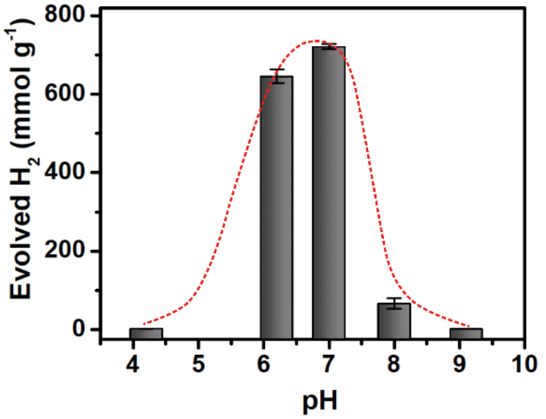

Figure S21. Photocatalytic data for binary Pdots in various pH range (from 4.2 till 9.1) in the presence of methyl viologen (5mM) and hydrogenase (158 pmol) and ascorbic acid (0.2 M, pH 4.2) or TEOA (20%, pH 6.2. 7, 8, 9) initiated by LED irradiation (50 mW·cm^{-2}, 420–750 nm). It should be noted that even though different sacrificial reagents were used to create desired pH (e.g. ascorbic acid and TEOA), the observed pH trends agree well with what has been previously reported using standard in vitro assays employing the reductant sodium dithionite (with and without the mediator methyl viologen).[14,15]

Table S5. The table comparing the performance of Pdots:H_2ase assembly with the related state-of-the art systems

**Photosensi-tezer**	**Catalyst**	**Conditions**	**Light mW/cm^2**	**TOF $mol_{H2}\cdot mol_{H2ase}^{-1}\cdot h^{-1}$**	**Activity $\mu mol_{H2}\cdot g_{cat}^{-1}\cdot h^{-1}$**	**EQE**	**Stability (h)**	**Ref.**
**P1/P2 Pdots**	[FeFe]-H_2ase	20% TEOA,<br>pH 7, 35 pmol H_2ase	LED (17W)<br>λ > 420 nm 50 mW/cm^2	4423	88460	1.1 %@405 n m	150	This Study
**Pdots based photosensitizers**								
PFTFQ-PtPy5<br>PFTFQ-PtPy15<br>PFTFQ-PtPy25<br>PFTFQ-PtIq5<br>PFTFQ-PtIq5<br>PFTFQ-PtIq5	Pt (5 mol. %)<br>Pt (15 mol. %)<br>Pt (25 mol. %)<br>Pt (5 mol. %)<br>Pt (15 mol. %)<br>Pt (25 mol. %)	Diethylamine	LED (20W)<br>λ > 420 nm	-	4100<br>12700<br>7700<br>4200<br>11100<br>1600	0.02@515 nm<br>0.4@515 nm<br>0.09@515 nm<br>0.05@515 nm<br>0.42@515 nm<br>0.03@515 nm	12	16
PFN-Br<br><br><br>PFNDPP-Br	Pt (1.5wt. %)<br>Pt (3wt. %)<br>Pt (5wt. %)<br>Pt (3wt. %)	TEOA<br><br><br>Ascorbic acid (0.2M), pH 4	Xe (300W),<br>λ > 300 nm<br><br>Xe (300W),<br>λ > 300 nm	-	4280<br>4600<br>2700<br>11160	N/A<br><br><br>0.44@650 nm	 <br><br>20	17
PF8DTBT	Pt (3wt. %)	Ascorbic acid (0.2 M), pH 4	Xe (300W)<br>λ (N/A)	-	3790	N/A	5	18
PCDTBT/<br>PC60BM	Pt (3wt. %)<br><br>Rh (3wt. %)<br>Au (3wt. %)<br>Cu (3wt. %)	Ascorbic acid (0.2 M)<br>Ascorbic acid (0.04 M)	Xe (300W)<br>λ > 420 nm	-	179000<br><br>~4000<br>~12000<br>~1000	3.72@420 nm	18	19
PFBT/PFODT BT/ITIC	Pt (6wt%)	Ascorbic acid (0.2 M), pH 4	LED (17W)<br>λ > 420 nm	-	60800	6.5@550 nm<br>7.1@600 nm	120	20
PTB7-Th/EH-IDTBR		Ascorbic acid (0.2 M), pH 4		-	64426	5.6@660 nm<br>6.2@700 nm	16	21
**Hydrogenase catalysts**								
**CDs**<br>Solvo-thermal ASP in EtOH	[FeFe]-H_2ase	10 % TEOA<br>pH 7	LED<br>λ > 420 nm 50 mW/cm^2	5220	600	1.7%@420 nm	168	22
**CDs**<br>SS Pyrolysis Citric acid+mod.	[NiFeSe]-H_2ase	0.1 M EDTA<br>pH 6.0	Xe lamp<br>AM 1.5 G<br>100mW/cm^2	3900	80	0.4%@360 nm	25	23
**5-carboxyeosin**	[FeFe]-H_2ase	0.04 M EDTA	Halogen lamps (λ >300 nm)<br>14.5mW/cm^2	1.98E+6	-	2.4% (whole used spectrum)	5	24
**CdTe** with Mercapto-propionic acid	[FeFe]-H_2ase	0.5 M Ascorbic acid<br>pH 4.75	Halogen lamp, AM 3 (150W)	90000	-	9%@532 nm	<1	25
**CdTe** with Mercapto-propionic acid	[NiFe]-H_2ase	0.05 M Ascorbic acid<br>pH 7.4	300 mW Nd:YLF laser (527 nm)	8280	300000	3.6%<br>(IQE)	1	26
**CdSe/CdS** nanorod	[NiFe]-H_2ase	100 mM cysteine<br>pH 7.35	4.04 mW<br>405 nm diode laser		$1.25\cdot 10^8$	52%@405<br>(IQE)	1	27
**N-doped TiO_2**	[FeFe]-H_2ase	0.025 M TEOA<br>pH 7.0	Sunlight<br>65 mW/cm^2	20500<br>(350 pmol)	-	-		28
**TiO_2+RuP**	[NiFeSe]-H_2ase	0.025 M EDTA<br>pH 7.0	Thungsten-halogen lamp (>420 nm)<br>45 mW/cm^2	40000<br>(50 pmol)	-	-	8	29
**Carbon nitride**	[NiFeSe]-H_2ase	0.1 M EDTA<br>pH 6.0	AM 1.5 G<br>100 mW/cm^2	5530<br>(50 pmol)	55	0.07%@365 n m<br>0.005%@465 nm	50	30

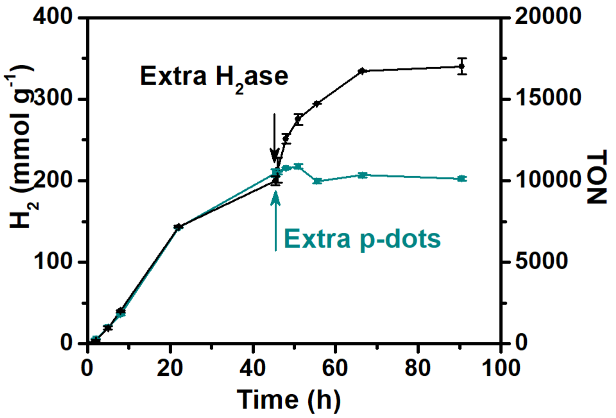

Figure S22. Addition of extra quantities of 50 µg polymer dots (a) or 150 pmol of hydrogenase (b) to a 10 vol% TEOA reaction mixture containing 5 mM methyl viologen and 158 pmol of hydrogenase (V_{total} = 2 mL) after 48 h of irradiation.

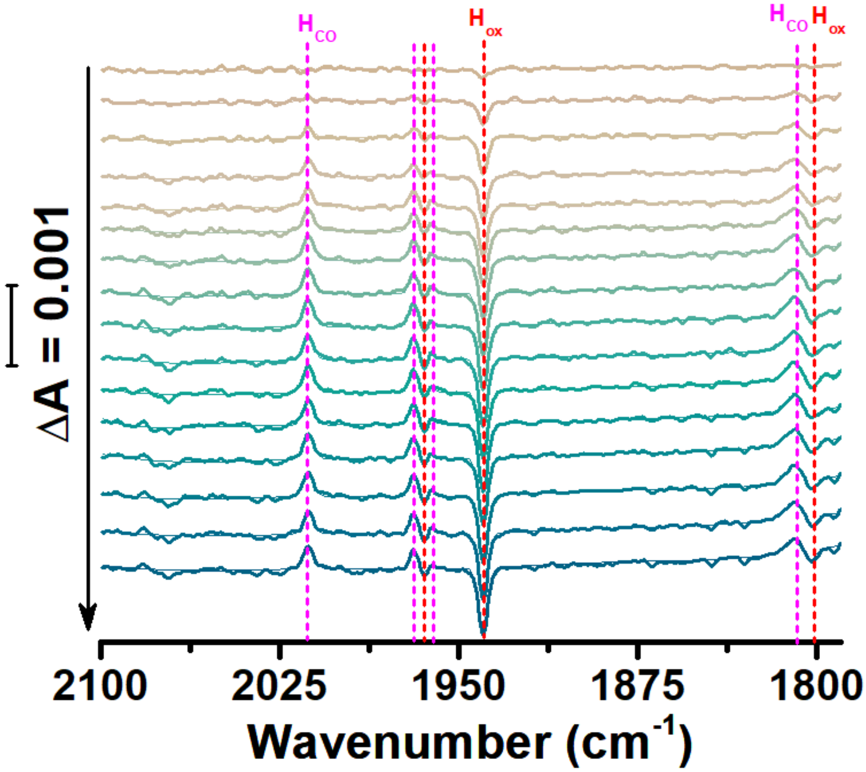

Figure S23. ATR-FTIR spectra of reaction mixture (Pdots 11 µg/ml, MV 3.9 mM, TEOA 6%, H_2ase 0.7 mM) during first minutes of LED illumination (50 mW·cm^{-2}, 420–750 nm).

IV. The effect of Pdots on the intrinsic oxygen tolerance of hydrogenase enzyme

To study the effect of Pdots on the intrinsic oxygen tolerance of hydrogenase enzyme, we have performed additional photocatalytic experiments in the presence of molecular O_2. Catalase was introduced in order to suppress the formation of reactive oxygen species (e.g. H_2O_2)[31,32] that are usually produced via reduction of O_2 by Pdots (**eq.S1**) and O_2 reduction by viologen mediator (**eq.S2-3**, *Chem. Soc. Rev.*, **1981**, *10*, 49–82). Reactive oxygen species cause degradation of photochemical systems and need to be avoided.[31]

$$Pdots + O_2 + H^+ \rightarrow Pdots^+ + H_2O_2 \; (eq.S1)$$

$$Pdots + MV^{2+} \leftrightarrow Pdots^+ + MV^{\bullet+} \; (eq.S2)$$

$$MV^{\bullet+} + O_2 + H^+ \rightarrow MV^{2+} + H_2O_2 \; (eq.S3)$$

$$2H_2O_2 \xrightarrow{Catalase} 2H_2O + O_2 \; (eq.S4)$$

$$\text{Net reaction: } 2Pdots + O_2 \xrightarrow{Catalase} 2Pdots^+ + 2H_2O \; (eq.S5)$$

The ability to reduce molecular oxygen by Pdots in the presence of sacrificial electron donor have been studied by Clark electrode. As seen from **Figure S24a** Pdots do indeed reduce molecular oxygen once the reaction mixture is illuminated. Introduction of catalase after 1000 seconds, leads to dismutation of H_2O_2 to yield water and 0.5 molar equivalents of O_2 (eq. S4). Photocatalytic experiments repeated in the presence of O_2 (up to 1% in the headspace), but without catalase revealed decline in initial rate of hydrogen evolution (**Figure S24b**). However, when catalase was present (**Figure S24b** green line), an increase in the initial rate of hydrogen formation was observed in respect to system without catalase. The overall activity of the biohybrid system in the presence of molecular O_2 in the headspace persisted for days and retained half of its initial activity for the system in the absence of O_2. In summary, combination of Pdots and catalase results in protection of hydrogenase enzyme against O_2 damage up to 0.3% in the headspace. In this case Pdots and methyl viologen successfully reduce molecular oxygen into H_2O_2, while the catalase facilitates removal of harmful ROS (H_2O_2).

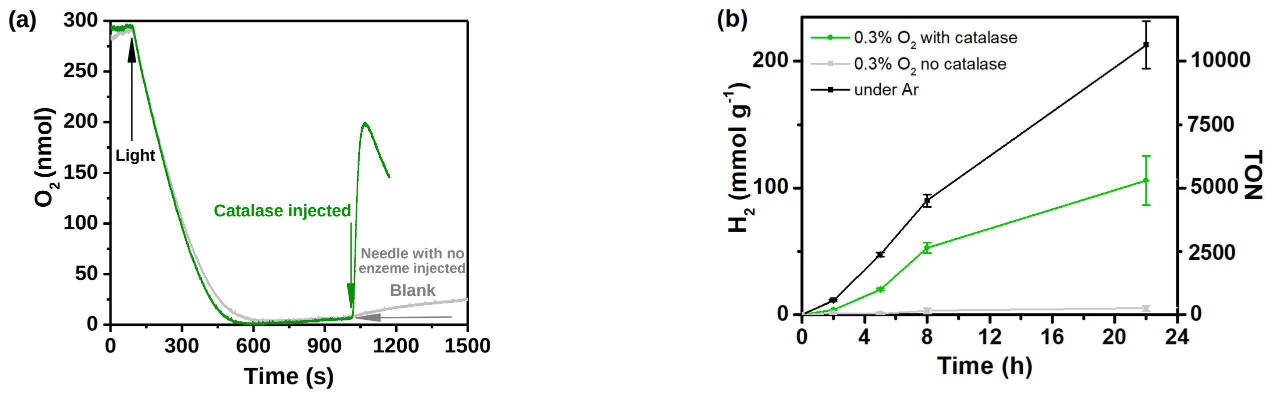

Figure S24 (a) O_2 reduction studies of P1/P2 Pdots in 19% TEOA under LED irradiation (50 mW/cm^2, 420–750 nm). After 1000 s catalase (green curve), or empty needle with no enzyme were introduced (light gray curve). (b) Photocatalysis of P1/P2 Pdots with catalase (green curve) and w/o catalase (light gray curve) in the presence of 0.3 % oxygen initially injected to the headspace before illumination (TEOA 19%, 5 mM methyl viologen, 158 pmol hydrogenase) at pH 7.

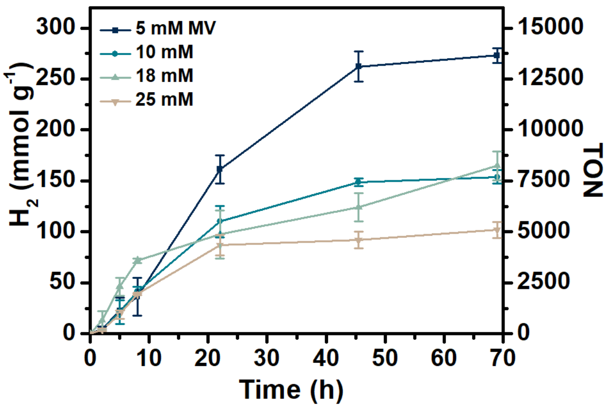

Figure S25. Hydrogen evolution data for polymer dots in the presence of various quantities of methyl viologen (5-25 mM) in 10 vol%, 0.67M TEOA with hydrogenase enzyme (158 pmol).

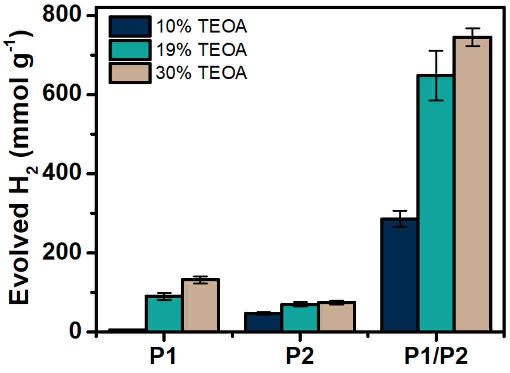

Figure S26. Photocatalytic data for Pdots based on single polymers P1 and P2 and for binary P1/P2 Pdots (blue line) in presence of various amount of TEOA (10% (dark blue column), 19% (cyan column), 30% (light brown-gray column), at pH 7), with methyl viologen (5mM) and hydrogenase (158 pmol) initiated by LED irradiation (50 mW·cm^{-2}, 420–750 nm).

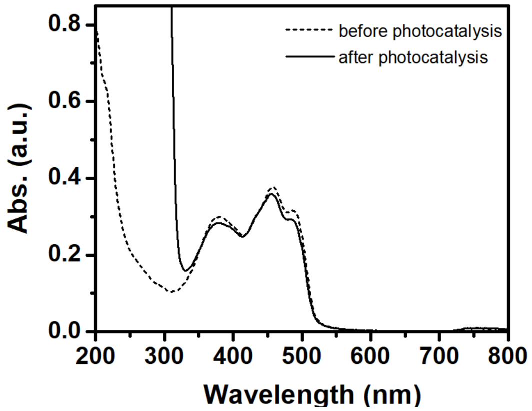

Figure S27. UV-Vis spectrum of Pdots before photocatalysis (dashed line) and UV-Vis spectrum of reaction mixture after photocatalysis during extended illumination (solid line).

References:

(1) Mulder, D. W.; Ortillo, D. O.; Gardenghi, D. J.; Naumov, A. V.; Ruebush, S. S.; Szilagyi, R. K.; Huynh, B. H.; Broderick, J. B.; Peters, J. W. Activation of HydAΔEFG Requires a Preformed [4Fe-4S] Cluster. *Biochemistry* **2009**, *48* (26), 6240–6248. https://doi.org/10.1021/bi9000563.

(2) Mészáros, L. S.; Németh, B.; Esmieu, C.; Ceccaldi, P.; Berggren, G. In Vivo EPR Characterization of Semi-Synthetic [FeFe] Hydrogenases. *Angew. Chem. Int. Ed.* **2018**, *57* (10), 2596–2599. https://doi.org/10.1002/anie.201710740.

(3) Park, S.; Baker, J. O.; Himmel, M. E.; Parilla, P. A.; Johnson, D. K. Cellulose Crystallinity Index: Measurement Techniques and Their Impact on Interpreting Cellulase Performance. *Biotechnol. Biofuels* **2010**, *3*. https://doi.org/10.1186/1754-6834-3-10.

(4) Pavlishchuk, V. V.; Addison, A. W. Conversion Constants for Redox Potentials Measured versus Different Reference Electrodes in Acetonitrile Solutions at 25°C. *Inorganica Chim. Acta* **2000**, *298* (1), 97–102. https://doi.org/10.1016/S0020-1693(99)00407-7.

(5) G. Scheblykin, I.; Yartsev, A.; Pullerits, T.; Gulbinas, V.; Sundström, V. Excited State and Charge Photogeneration Dynamics in Conjugated Polymers. *J. Phys. Chem. B* **2007**, *111* (23), 6303–6321. https://doi.org/10.1021/jp068864f.

(6) Kosco, J.; Gonzalez-Carrero, S.; Howells, C. T.; Zhang, W.; Moser, M.; Sheelamanthula, R.; Zhao, L.; Willner, B.; Hidalgo, T. C.; Faber, H.; Purushothaman, B.; Sachs, M.; Cha, H.; Sougrat, R.; Anthopoulos, T. D.; Inal, S.; Durrant, J. R.; McCulloch, I. Oligoethylene Glycol Side Chains Increase Charge Generation in Organic Semiconductor Nanoparticles for Enhanced Photocatalytic Hydrogen Evolution. *Adv. Mater.* **2021**, 2105007. https://doi.org/https://doi.org/10.1002/adma.202105007.

(7) Banerji, N.; Cowan, S.; Leclerc, M.; Vauthey, E.; J. Heeger, A. Exciton Formation, Relaxation, and Decay in PCDTBT. *J. Am. Chem. Soc.* **2010**, *132* (49), 17459–17470. https://doi.org/10.1021/ja105290e.

(8) Ravirajan, P.; Haque, S. A.; Poplavskyy, D.; Durrant, J. R.; Bradley, D. D. C.; Nelson, J. Nanoporous TiO2 Solar Cells Sensitised with a Fluorene-Thiophene Copolymer. *Thin Solid Films* **2004**, *451–452*, 624–629. https://doi.org/10.1016/j.tsf.2003.11.031.

(9) Ravirajan, P.; Haque, S. A.; Durrant, J. R.; Poplavskyy, S. A.; Bradley, D. D. C.; Nelson, J. Hybrid Nanocrystalline TiO2 Solar Cells with a Fluorene-Thiophene Copolymer as a Sensitizer and Hole Conductor. *J. Appl. Phys.* **2004**, *95* (3), 1473. https://doi.org/10.1063/1.1638614.

(10) Yonezava, K.; Ito, M.; Kamioka, H.; Yasuda, T.; Han, L.; Moritomo, Y. Carrier Formation Dynamics of Organic Photovoltaics as Investigated by Time-Resolved Spectroscopy. *Adv. Opt. Technol.* **2012**, *2012*, 316045. https://doi.org/10.1155/2012/316045.

(11) Moritomo, Y.; Yasuda, T.; Yonezawa, K.; Sakurai, T.; Takeichi, Y.; Suga, H.; Takahashi, Y.; Inami, N.; Mase, K.; Ono, K. Fullerene Mixing Effect on Carrier Formation in Bulk-Hetero Organic Solar Cell. *Sci. Rep.* **2015**, *5*, 9483. https://doi.org/10.1038/srep09483.

(12) Banerji, N. Sub-Picosecond Delocalization in the Excited State of Conjugated Homopolymers and Donor-Acceptor Copolymers. *J. Mater. Chem. C* **2013**, *1*, 3052–3066. https://doi.org/10.1039/c3tc00005b.

(13) Jespersen, K. G.; Beenken, W. J. D.; Zaushitsyn, Y.; Yartsev, A.; Andersson, M.; Pullerits, T.; Sundström, V. The Electronic States of Polyfluorene Copolymers with Alternating Donor-Acceptor Units. *J. Chem. Phys.* **2004**, *121* (24), 12613. https://doi.org/10.1063/1.1817873.

(14) Happe, T.; Naber, J. D. Isolation, Characterization and N-terminal Amino Acid Sequence of Hydrogenase from the Green Alga Chlamydomonas Reinhardtii. *Eur. J. Biochem.* **1993**, *214* (2), 475–481. https://doi.org/10.1111/j.1432-1033.1993.tb17944.x.

(15) Senger, M.; Mebs, S.; Duan, J.; Shulenina, O.; Laun, K.; Kertess, L.; Wittkamp, F.; Apfel, U. P.; Happe, T.; Winkler, M.; Haumann, M.; Stripp, S. T. Protonation/Reduction Dynamics at the [4Fe-4S] Cluster of the Hydrogen-Forming Cofactor in [FeFe]-Hydrogenases. *Phys. Chem. Chem. Phys.*

2018, *20* (5), 3128–3140. https://doi.org/10.1039/c7cp04757f.
(16) Tseng, P.-J. J.; Chang, C.-L. L.; Chan, Y.-H. H.; Ting, L.-Y. Y.; Chen, P.-Y. Y.; Liao, C.-H. H.; Tsai, M.-L. L.; Chou, H.-H. H. Design and Synthesis of Cycloplatinated Polymer Dots as Photocatalysts for Visible-Light-Driven Hydrogen Evolution. *ACS Catal.* **2018**, *8* (9), 7766–7772. https://doi.org/10.1021/acscatal.8b01678.
(17) Hu, Z.; Zhang, X.; Yin, Q.; Liu, X.; Jiang, X. fang; Chen, Z.; Yang, X.; Huang, F.; Cao, Y. Highly Efficient Photocatalytic Hydrogen Evolution from Water-Soluble Conjugated Polyelectrolytes. *Nano Energy* **2019**, *60*, 775–783. https://doi.org/10.1016/j.nanoen.2019.04.027.
(18) Zhang, X.; Shen, F.; Hu, Z.; Wu, Y.; Tang, H.; Jia, J.; Wang, X.; Huang, F.; Cao, Y. Biomass Nanomicelles Assist Conjugated Polymers/Pt Cocatalysts to Achieve High Photocatalytic Hydrogen Evolution. *ACS Sustain. Chem. Eng.* **2019**, *7* (4), 4128–4135. https://doi.org/10.1021/acssuschemeng.8b05637.
(19) Yang, H.; Li, X.; Sprick, R. S.; Cooper, A. I.; Sebastian Sprick, R.; Cooper, A. I. Conjugated Polymer Donor-Molecular Acceptor Nanohybrids for Photocatalytic Hydrogen Evolution: Conjugated Polymer Donor-Molecular Acceptor Nanohybrids for Photocatalytic Hydrogen Evolution. *Chem. Commun.* **2020**, *56*, 6790–6793. https://doi.org/10.26434/chemrxiv.11418375.v1.
(20) Liu, A.; Gedda, L.; Axelsson, M.; Pavliuk, M.; Edwards, K.; Hammarström, L.; Tian, H. Panchromatic Ternary Polymer Dots Involving Sub-Picosecond Energy and Charge Transfer for Efficient and Stable Photocatalytic Hydrogen Evolution. *J. Am. Chem. Soc.* **2021**, *143* (7), 2875–2885. https://doi.org/10.1021/jacs.0c12654.
(21) Kosco, J.; Bidwell, M.; Cha, H.; Martin, T.; Howells, C. T.; Sachs, M.; Anjum, D. H.; Gonzalez Lopez, S.; Zou, L.; Wadsworth, A.; Zhang, W.; Zhang, L.; Tellam, J.; Sougrat, R.; Laquai, F.; DeLongchamp, D. M.; Durrant, J. R.; McCulloch, I. Enhanced Photocatalytic Hydrogen Evolution from Organic Semiconductor Heterojunction Nanoparticles. *Nat. Mater.* **2020**, *19* (5), 559–565. https://doi.org/10.1038/s41563-019-0591-1.
(22) Holá, K.; V. Pavliuk, M.; Németh, B.; Huang, P.; Zdražil, L.; Land, H.; Berggren, G.; Tian, H. Carbon Dots and [FeFe] Hydrogenase Biohybrid Assemblies for Efficient Light-Driven Hydrogen Evolution. *ACS Catal.* **2020**, *10* (17), 9943–9952. https://doi.org/10.1021/acscatal.0c02474.
(23) Hutton, G. A. M.; Reuillard, B.; Martindale, B. C. M.; Caputo, C. A.; Lockwood, C. W. J.; Butt, J. N.; Reisner, E. Carbon Dots as Versatile Photosensitizers for Solar-Driven Catalysis with Redox Enzymes. *J. Am. Chem. Soc.* **2016**, *138* (51), 16722–16730. https://doi.org/10.1021/jacs.6b10146.
(24) Adam, D.; Bösche, L.; Castañeda-Losada, L.; Winkler, M.; Apfel, U.-P.; Happe, T. Sunlight-Dependent Hydrogen Production by Photosensitizer/Hydrogenase Systems. *ChemSusChem* **2017**, *10* (5), 894–902. https://doi.org/10.1002/cssc.201601523.
(25) Brown, K. A.; Dayal, S.; Ai, X.; Rumbles, G.; King, P. W. Controlled Assembly of Hydrogenase-CdTe Nanocrystal Hybrids for Solar Hydrogen Production. *J. Am. Chem. Soc.* **2010**, *132* (28), 9672–9680. https://doi.org/10.1021/ja101031r.
(26) L. Greene, B.; A. Joseph, C.; J. Maroney, M.; Brian Dyer, R. Direct Evidence of Active-Site Reduction and Photodriven Catalysis in Sensitized Hydrogenase Assemblies. *J. Am. Chem. Soc.* **2012**, *134* (27), 11108–11111. https://doi.org/10.1021/ja3042367.
(27) Chica, B.; Wu, C. H.; Liu, Y.; Adams, M. W. W.; Lian, T.; Dyer, R. B. Balancing Electron Transfer Rate and Driving Force for Efficient Photocatalytic Hydrogen Production in CdSe/CdS Nanorod-[NiFe] Hydrogenase Assemblies. *Energy Environ. Sci.* **2017**, *10* (10), 2245–2255. https://doi.org/10.1039/c7ee01738c.
(28) Polliotto, V.; Morra, S.; Livraghi, S.; Valetti, F.; Gilardi, G.; Giamello, E. Electron Transfer and H2 Evolution in Hybrid Systems Based on [FeFe]-Hydrogenase Anchored on Modified TiO2. *Int. J. Hydrogen Energy* **2016**, *41* (25), 10547–10556. https://doi.org/10.1016/j.ijhydene.2016.05.002.
(29) Reisner, E.; Fontecilla-Camps, J. C.; Armstrong, F. A. Catalytic Electrochemistry of a [NiFeSe]-Hydrogenase on TiO2 and Demonstration of Its Suitability for Visible-Light Driven H2 Production.

Chem. Commun. **2009**, No. 5, 550–552. https://doi.org/10.1039/b817371k.
(30) Caputo, C. a.; Gross, M. a.; Lau, V. W.; Cavazza, C.; Lotsch, B. V.; Reisner, E. Photocatalytic Hydrogen Production Using Polymeric Carbon Nitride with a Hydrogenase and a Bioinspired Synthetic Ni Catalyst. *Angew. Chem. Int. Ed.* **2014**, *53*, 11538–11542. https://doi.org/10.1002/anie.201406811.
(31) Li, H.; Münchberg, U.; Oughli, A. A.; Buesen, D.; Lubitz, W.; Freier, E.; Plumeré, N. Suppressing Hydrogen Peroxide Generation to Achieve Oxygen-Insensitivity of a [NiFe] Hydrogenase in Redox Active Films. *Nat. Commun.* **2020**, *11* (1), 920. https://doi.org/10.1038/s41467-020-14673-7.
(32) Ruff, A.; Szczesny, J.; Marković, N.; Conzuelo, F.; Zacarias, S.; Pereira, I. A. C.; Lubitz, W.; Schuhmann, W. A Fully Protected Hydrogenase/Polymer-Based Bioanode for High-Performance Hydrogen/Glucose Biofuel Cells. *Nat. Commun.* **2018**, *9* (1), 3675. https://doi.org/10.1038/s41467-018-06106-3.
